# Extreme differences in SARS-CoV-2 viral loads among respiratory specimen types during presumed pre-infectious and infectious periods

**DOI:** 10.1093/pnasnexus/pgad033

**Published:** 2023-03-14

**Authors:** Alexander Viloria Winnett, Reid Akana, Natasha Shelby, Hannah Davich, Saharai Caldera, Taikun Yamada, John Raymond B Reyna, Anna E Romano, Alyssa M Carter, Mi Kyung Kim, Matt Thomson, Colten Tognazzini, Matthew Feaster, Ying-Ying Goh, Yap Ching Chew, Rustem F Ismagilov

**Affiliations:** California Institute of Technology, 1200 E. California Blvd, Pasadena, CA 91125, USA; California Institute of Technology, 1200 E. California Blvd, Pasadena, CA 91125, USA; California Institute of Technology, 1200 E. California Blvd, Pasadena, CA 91125, USA; California Institute of Technology, 1200 E. California Blvd, Pasadena, CA 91125, USA; California Institute of Technology, 1200 E. California Blvd, Pasadena, CA 91125, USA; Pangea Laboratory LLC, 14762 Bentley Cir, Tustin, CA 92780, USA; Zymo Research Corp., 17062 Murphy Ave, Irvine, CA 92614, USA; Pangea Laboratory LLC, 14762 Bentley Cir, Tustin, CA 92780, USA; California Institute of Technology, 1200 E. California Blvd, Pasadena, CA 91125, USA; California Institute of Technology, 1200 E. California Blvd, Pasadena, CA 91125, USA; California Institute of Technology, 1200 E. California Blvd, Pasadena, CA 91125, USA; California Institute of Technology, 1200 E. California Blvd, Pasadena, CA 91125, USA; Pasadena Public Health Department, 1845 N. Fair Oaks Ave, Pasadena, CA 91103, USA; Pasadena Public Health Department, 1845 N. Fair Oaks Ave, Pasadena, CA 91103, USA; Pasadena Public Health Department, 1845 N. Fair Oaks Ave, Pasadena, CA 91103, USA; Pangea Laboratory LLC, 14762 Bentley Cir, Tustin, CA 92780, USA; Zymo Research Corp., 17062 Murphy Ave, Irvine, CA 92614, USA; California Institute of Technology, 1200 E. California Blvd, Pasadena, CA 91125, USA

**Keywords:** COVID-19, testing strategies, viral loads

## Abstract

SARS-CoV-2 viral-load measurements from a single-specimen type are used to establish diagnostic strategies, interpret clinical-trial results for vaccines and therapeutics, model viral transmission, and understand virus–host interactions. However, measurements from a single-specimen type are implicitly assumed to be representative of other specimen types. We quantified viral-load timecourses from individuals who began daily self-sampling of saliva, anterior-nares (nasal), and oropharyngeal (throat) swabs before or at the incidence of infection with the Omicron variant. Viral loads in different specimen types from the same person at the same timepoint exhibited extreme differences, up to 10^9^ copies/mL. These differences were not due to variation in sample self-collection, which was consistent. For most individuals, longitudinal viral-load timecourses in different specimen types did not correlate. Throat-swab and saliva viral loads began to rise as many as 7 days earlier than nasal-swab viral loads in most individuals, leading to very low clinical sensitivity of nasal swabs during the first days of infection. Individuals frequently exhibited presumably infectious viral loads in one specimen type while viral loads were low or undetectable in other specimen types. Therefore, defining an individual as infectious based on assessment of a single-specimen type underestimates the infectious period, and overestimates the ability of that specimen type to detect infectious individuals. For diagnostic COVID-19 testing, these three single-specimen types have low clinical sensitivity, whereas a combined throat–nasal swab, and assays with high analytical sensitivity, was inferred to have significantly better clinical sensitivity to detect presumed pre-infectious and infectious individuals.

**Significance Statement**

In a longitudinal study of SARS-CoV-2 Omicron viral loads in three paired specimen types (saliva, anterior-nares swabs, and oropharyngeal swabs), we found extreme differences among paired specimen types collected from a person at the same timepoint, and that viral loads in different specimen types from the same person often do not correlate throughout infection. Individuals often exhibited high, presumably infectious viral loads in oral specimen types before nasal viral loads remained low or even undetectable. Combination nasal–throat swabs were inferred to have superior clinical sensitivity to detect infected and infectious individuals. This demonstrates that single-specimen type reference standard tests for SARS-CoV-2, such as in clinical trials or diagnostics evaluations may miss infected and even infectious individuals.

## Introduction

Measurements of viral load in respiratory infections are used to establish diagnostic strategies, interpret results of clinical trials of vaccines and therapeutics, model viral transmission, and understand virus–host interactions. But how viral loads change across multiple specimen types early in SARS-CoV-2 infection is not well understood. Specifically in the context of diagnostics, as new SARS-CoV-2 variants-of-concern (and new respiratory viruses) emerge with different viral kinetics (1), it is imperative to continually re-evaluate testing strategies (including specimen type and test analytical sensitivity) for detecting pre-infectious and infectious individuals. Early detection can reduce transmission within communities (2, 3) and the global spread of new variants, and enable earlier initiation of treatment resulting in better outcomes (4–6).

Selecting testing strategies to achieve detection in the pre-infectious and infectious periods requires filling two critical knowledge gaps: (i) Which respiratory specimen type accumulates virus first? (ii) What is the appropriate test analytical sensitivity to detect accumulation of virus in the pre-infectious and infectious stages? These two gaps must be filled in parallel. Commonly, an individual's infection is described by the viral load sampled from a single-specimen type, which is appropriate when there is one principal specimen type (e.g. HIV in blood plasma). However, some respiratory pathogens, including SARS-CoV-2, can infect multiple respiratory sampling sites (7–9).

Nasopharyngeal swabs have been the gold standard for SARS-CoV-2 detection but are poorly tolerated and challenging for serial sampling and self-collection. Many alternate specimen types are now widely used. Some of these are suitable for routine testing, and are approved for self-collection (e.g. saliva, anterior-nares [nasal] swabs, and oropharyngeal [throat] swabs) in some countries. Cross-sectional studies comparing paired specimen types from the same person have shown that cycle threshold (Ct, a semi-quantitative proxy for viral load) values can differ substantially between specimen types (10), and the clinical sensitivity of different specimen types is not equivalent (11). Sometimes, viral loads in one specimen type are low or even absent while viral loads in another type are high (12–14). Nasal swabs (including those used for rapid antigen testing) are the dominant specimen type used in the United States for workplace screenings and at-home testing. However, several studies (15–18), news media (19), and social-media posts have speculated that in Omicron infections, viral load accumulates in oral specimens before the nasal cavity. Formal investigations of specimen types from single timepoints and cross-sectional studies have been contradictory, potentially due to when individuals were sampled; viral loads from individuals sampled after symptom onset may not reflect viral loads from earlier in the infection. Rigorous, longitudinal comparisons of paired specimen types starting from the incidence of infection are needed to fill this gap.

The second knowledge gap is the analytical sensitivity needed for reliable detection of pre-infectious and infectious individuals. The assay analytical sensitivity is described by the limit of detection (LOD); generally, the LOD of an assay describes its ability to detect and quantify target at or above a certain concentration in that specimen type with >95% probability (20). Assays with high LODs (low-analytical sensitivity) require a high concentration of virus to reliably yield positive results, whereas assays with low LODs (high analytical sensitivity) can reliably detect much lower concentrations of virus. For example, in early SARS-CoV-2 variants, some studies showed that saliva accumulated virus earlier than nasal swabs, but at low levels (14, 21, 22), thus saliva required a high-analytical-sensitivity (low LOD) assay (14, 23). However, low-analytical-sensitivity tests (including rapid antigen tests) are increasingly authorized and used globally (24, 25). Which of these tests can detect pre-infectious and infectious individuals requires quantitative, longitudinal measurements of viral concentration in multiple specimen types starting from the incidence of infection.

Early detection, in the pre-infectious period, is ideal to prompt infection-control practices (e.g. isolation) before transmission occurs, and detection during the infectious period is critical to minimize outbreaks. Replication-competent (i.e. infectious) virus has been recovered from saliva (9), oropharyngeal swabs (26), and nasal swabs (27), but it is impractical and infeasible to perform viral culture on each positive specimen to determine if a person is infectious. However, studies that performed both culture and RT-qPCR found that low Ct values (high viral loads) are associated with infectious virus. Specific viral loads likely to be infectious for each specimen type have not been established (28), partly because Ct values are not comparable across assays (29, 30) and culture methods differ. However, as a general reference, viral loads of >10^4^–10^7^ RNA copies/mL are associated with the presence of replication-competent virus (17, 31–41), and these values have been used in outbreak simulations (35, 39, 42–44). The enormous range (>4 orders of magnitude) in observed viral loads that correspond with infectiousness emphasizes why quantitative measurements of loads in different specimen types are needed to make robust predictions about tests that will detect the pre-infectious and infectious periods.

The assumption made early in the COVID-19 pandemic that viral load always rises rapidly from undetectable to likely infectious (45) has been challenged by numerous longitudinal studies of viral load in different specimen types that show early SARS-CoV-2 viral loads can rise slowly over days (14, 17, 18, 21, 27, 41, 46–49), not hours. These findings are encouraging because a longer window provides more time to identify and isolate pre-infectious individuals. However, making use of this opportunity by selecting an optimal diagnostic test requires a thorough understanding of how viral load changes in each specimen type early in infection. Moreover, to reliably detect an infectious person, the infectious specimen must be tested with an assay that has an LOD below the infectious viral load for that specimen type. However, many authorized COVID-19 tests (including rapid antigen tests) have LODs well above the range of reported infectious viral loads (50, 51).

Filling the two critical and inter-related knowledge gaps about specimen type and assay LOD requires high-frequency quantification of viral loads, rather than semi-quantitative Ct values, in multiple specimen types starting from the incidence of infection, not after a positive test or after symptom onset, as is commonly done. Moreover, quantification must be performed with a high-analytical-sensitivity assay to capture low viral loads in the first days of detectable infection. It is challenging to acquire such data. Individuals at high risk of infection must be prospectively enrolled prior to detectable infection and tested longitudinally with high-frequency in multiple paired specimen types.

To our knowledge, four studies have reported longitudinal viral-load timecourses in multiple, paired specimen types from early infection. A university study (27) captured daily saliva and nasal-swab samples for 2 weeks from 60 individuals, only 3 of whom were negative for SARS-CoV-2 upon enrollment. In our prior study, we captured twice-daily viral-load timecourses from 72 individuals for 2 weeks (52), 7 of whom were negative upon enrollment (14). In six of seven individuals, we inferred from viral-load quantifications that a high-analytical-sensitivity saliva assay would detect infections earlier than a low-analytical-sensitivity nasal-swab test. In a SARS-CoV-2 human challenge study (17), 10 of 18 infected participants had detectable virus by PCR in throat swabs at least 1 day prior to nasal swabs, and replication-competent virus was recovered from throat swabs before nasal swabs in at least 12 of 18 participants. Participants in these three studies were infected with pre-Omicron variants. One longitudinal study (15) analyzed viral loads in saliva, nasal swabs, and throat swabs in Omicron; however, daily measurements in all three specimen types were captured for only two individuals, both of whom were already positive upon enrollment. A separate case-ascertained household-transmission study with prospective daily sampling also captured viral-load measurements from the incidence of infection using a combination nasal–throat swab specimen type (41). In the United Kingdom, where this study was performed, a combination nasal–throat swab specimen type is regularly used for diagnostic testing (53, 54). However, the rise and fall of Omicron viral loads in multiple paired single-specimen types from the incidence of infection has not been characterized, despite these data being necessary to define the appropriate test analytical sensitivity and specimen type to best detect pre-infectious and infectious individuals.

Here, we measured and analyzed the viral-load timecourses of the Omicron variant in three specimen types appropriate for self-sampling (saliva, nasal swabs, and throat swabs) by individuals starting at or before the incidence of infection as part of a household-transmission study in Southern California. We then utilized these data to determine which specimen type and analytical sensitivity would yield the most reliable detection of pre-infectious and infectious individuals. A separate paper reports the results of daily rapid antigen testing in this study (55).

## Materials and methods

### Study design

This case-ascertained study of household transmission (approved under Caltech IRB #20-1026) was conducted in the greater Los Angeles County area between November 23, 2021, and March 1, 2022. All adult participants provided written informed consent; all minor participants provided verbal assent accompanied by written permission from a legal guardian. Children aged 8–17 years old additionally provided written assent. See Supplemental Information for details.

**Fig. 1.**
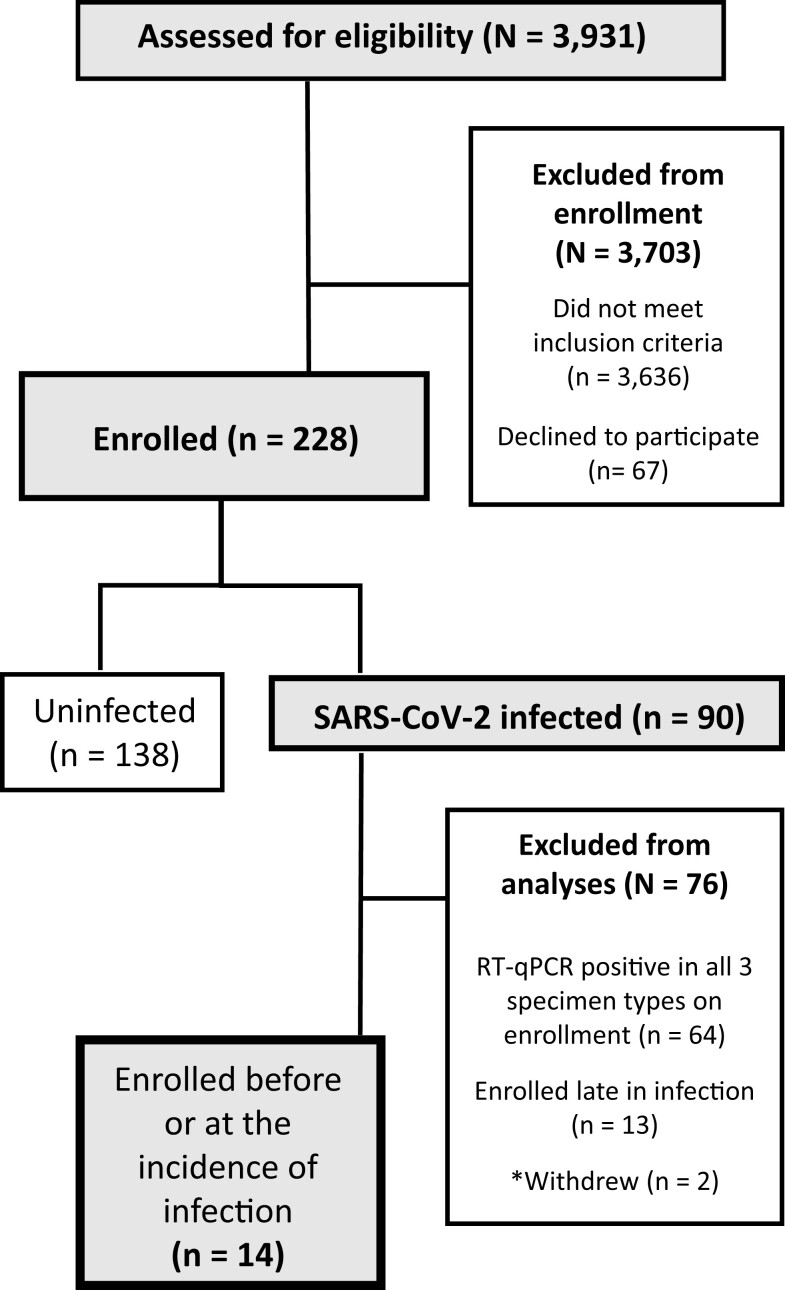
A CONSORT diagram shows participant recruitment, eligibility, enrollment, and selection for inclusion in the study cohort.

**Fig. 2.**
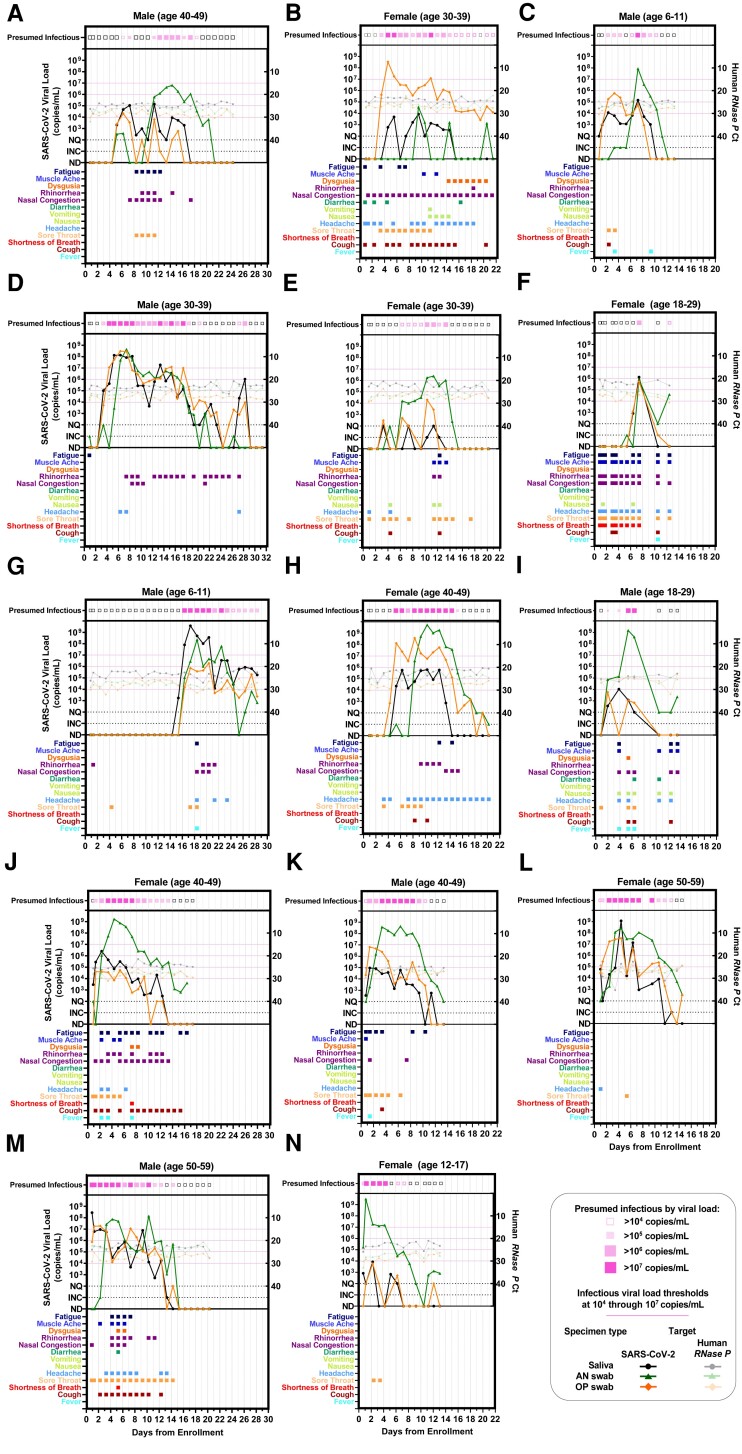
Individual viral-load timecourse measurements from 14 participants enrolled at or before the incidence of acute SARS-CoV-2 infection. Each panel (A–N) represents a single participant throughout the course of enrollment. Each panel plots SARS-CoV-2 viral-load measurements (left *y*-axis) and human *RNase P* Ct values (right *y*-axis). Line colors indicate specimen type: black/grey circles are saliva, green triangles are anterior-nares (AN) swabs, and orange diamonds are oropharyngeal (OP) swabs. Timepoints at which at least one specimen type had presumably infectious viral load (>10^4^–10^7^ copies/mL) are indicated at the top of each plot. Colored boxes below each plot indicate the symptoms reported at each sample-collection timepoint. Each of the 14 participants collected three specimen types throughout the course of acute infection, resulting in 42 viral-load timecourses. Participants collected an average of 15 (±5 SD) daily timepoints. ND, not detected; INC, inconclusive result; NQ, virus detected, however, viral loads below the test LOD (250 copies/mL) and thus not reliably quantifiable for RT-qPCR measurements.

A total of 228 participants from 56 households were enrolled; 90 of whom tested positive for SARS-CoV-2 infection during enrollment (Fig. 1). We limited our analyses to 14 individuals (Tables S1 and S5, Fig. 2) who enrolled in the study at or before the incidence of acute SARS-CoV-2 infection. To be included in the cohort, a participant must have had at least one specimen type with viral loads below quantification upon enrollment, followed by positivity and quantifiable viral loads in all three specimen types.

Each day, participants reported symptoms, then self-collected saliva, anterior-nares (nasal) swab, and posterior oropharyngeal (hereafter throat) swab specimens for RT-qPCR testing in Zymo Research SafeCollect devices (CE-marked for EU use), following manufacturer's instructions (56, 57). Participants collected specimens immediately upon enrollment, then daily upon waking, as morning sample collection has been shown to yield higher viral loads than evening collection (52).

### RT-qPCR testing for SARS-CoV-2

Extraction and RT-qPCR were performed at Pangea Laboratories (Tustin, CA, USA) using the FDA-authorized *Quick* SARS-CoV-2 RT-qPCR kit, with results assigned per manufacturer criteria (58). Additional details in Supplemental Information. This assay has a reported LOD of 250 copies/mL of sample.

### Quantification of viral load from RT-qPCR result

To quantify viral load in RT-qPCR specimens, contrived specimens across a 13-point standard curve (dynamic range from 250 to 4.50 × 10^8^ copies/mL) for each specimen type was generated at Caltech and underwent extraction and RT-qPCR as described above. All three replicates at 250 copies/mL of specimen were detected, independently validating the reported LOD for the assay. For each specimen type, the standard curve generated an equation to convert from SARS-CoV-2 *N* gene Ct values to viral loads in genomic copy equivalents (hereafter copies) per mL of each specimen type. See Supplemental Information for additional details and equations. Positive specimens with viral loads that would be quantified below the assay LOD were considered not quantifiable.

### Viral sequencing and lineage/variant determination

Viral sequencing of at least one specimen for each participant with incident infection was performed on nasal or throat specimens with moderate to high viral loads by Zymo Research at Pangea Lab. See Supplemental Information for details.

### Defining pre-infectious and infectious periods

The pre-infectious period is all SARS-CoV-2-positive timepoints prior to the first timepoint in which any specimen type contains viral load greater than the indicated infectious viral-load threshold. There are three main methods for defining the infectious period for an individual based on viral loads. First, the infectious period may be defined as the continuous period between the first specimen (of any type) with an infectious viral load until the first timepoint after which no specimen has an infectious viral load (59, 60). Or, to account for viral-load fluctuations, one may instead define an instantaneous infectious period (i.e. an individual is presumed infectious only when at least one specimen type has a viral load above the infectious viral load threshold). Both methods neglect the role of the neutralizing immune response, and the impact of infection stage on viral-culture positivity (32, 61, 62). To account for these factors, the infectious period may be limited to a number of days following symptoms or the first infectious timepoint. Our analyses (Table S3, Fig. 7), include all three common definitions. First, we used a “continuous infectious period” whereby a participant is presumed infectious for all timepoints between the first specimen with an infectious viral load and the first timepoint after which no specimens had infectious viral loads. Second, we used an “instantaneous infectious period,” which presumes that a participant is infectious only at timepoints when viral load in at least one specimen type is above the infectious viral load threshold. Third, we presumed that a participant is infectious only for the first 5 days from their first timepoint when viral load in at least one specimen type rose above the infectious viral-load threshold.

**Fig. 3.**
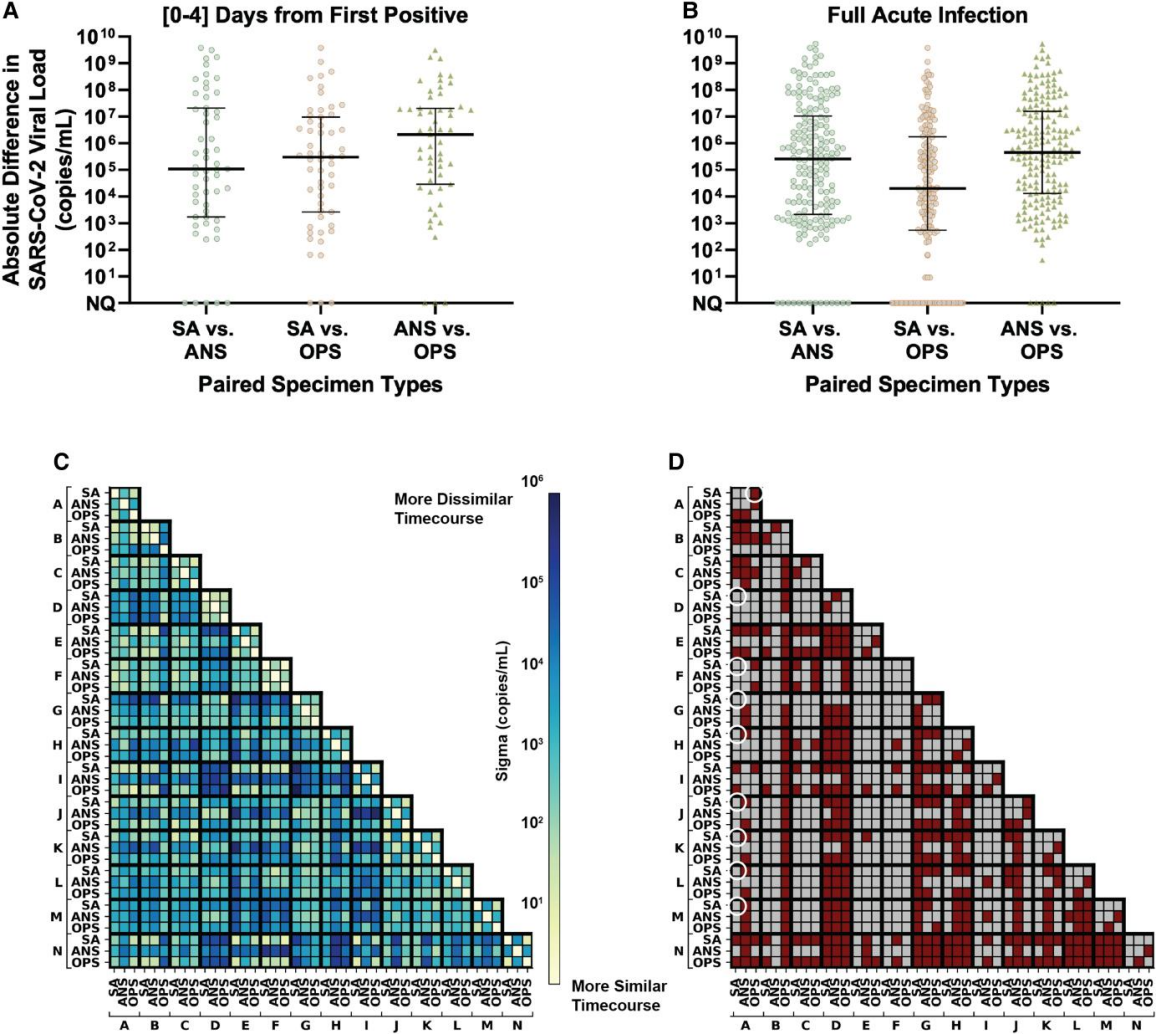
Extreme differences in viral loads across specimen types collected from the same person at the same timepoint for the 14 participants enrolled before or at the incidence of acute SARS-CoV-2 infection. A, B) Absolute differences in viral loads across paired specimen types were calculated as the absolute value of viral load in one specimen type minus another from the same participant at the same specimen-collection timepoint. Black lines indicate median, with interquartile range. Differences are shown for: A) 55 timepoints collected in the first 4 days from the incidence of infection (first positive specimen of any type) in each participant and B) 186 timepoints collected throughout the entirety of acute infection (at least one specimen type from the participant at the timepoint was positive and had quantifiable SARS-CoV-2 viral load; 11 timepoints were positive but not quantifiable). C) Correlation of viral-load timecourses, measured as the standard deviation across paired viral-load timecourses, assuming Gaussian-distributed noise (see Methods “Comparison of Viral-Load Timecourses Across Specimen Types”). D) Statistical significance of the difference in viral-load timecourses between specimens and between participants. Statistically significantly different timecourses are represented as red cells and nonsignificant comparisons are grey. White circles are called out as examples in the text. Expected sampling noise was estimated by analyzing *RNase P* Ct data from our study (Fig. S4) and from Levy et al. (63). *P*-values were obtained by comparing residuals from observed data and expected sampling noise. Additional method details are shown in Fig. S5. SA, saliva; ANS, anterior-nares nasal swab; OPS, oropharyngeal swab. Participant labels match Fig. 2 panels (A–N).

**Fig. 4.**
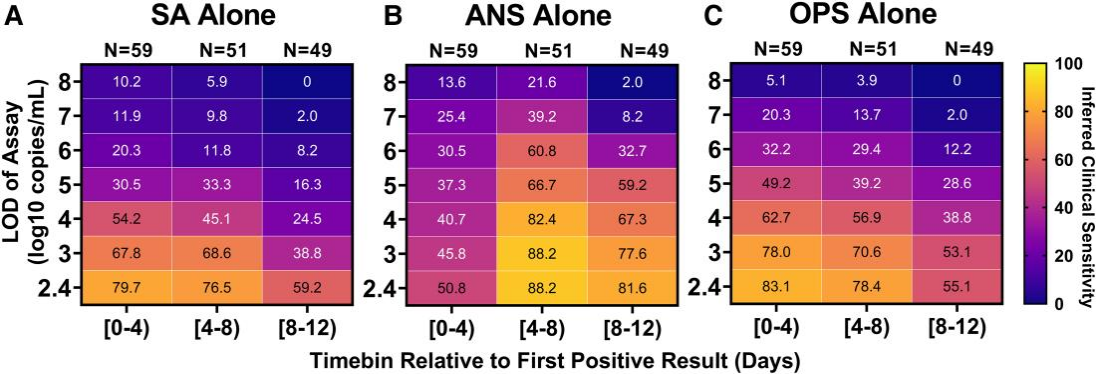
Inferred clinical sensitivity of assays with different LODs to detect infected persons by any single specimen type (A–C). Heatmaps show the inferred clinical sensitivity as a function of test LOD throughout the course of the infection (in 4-day timebins relative to the first positive specimen of any type) for (A) SA specimens alone, (B) nasal-swab specimens alone, and (C) throat-swab specimens alone. Inferred clinical sensitivity was calculated as the number of specimens of the given type with viral loads greater than the given LOD divided by the total number of specimens collected within that timebin. *N* indicates the number of timepoints. Only timepoints where at least one specimen type had a quantifiable viral load (≥250 copies/mL) were included. Two-day timebins are shown in Fig. S6. The performances of computationally contrived combination specimen types are shown in Figs. S6 and S7.

**Fig. 5.**
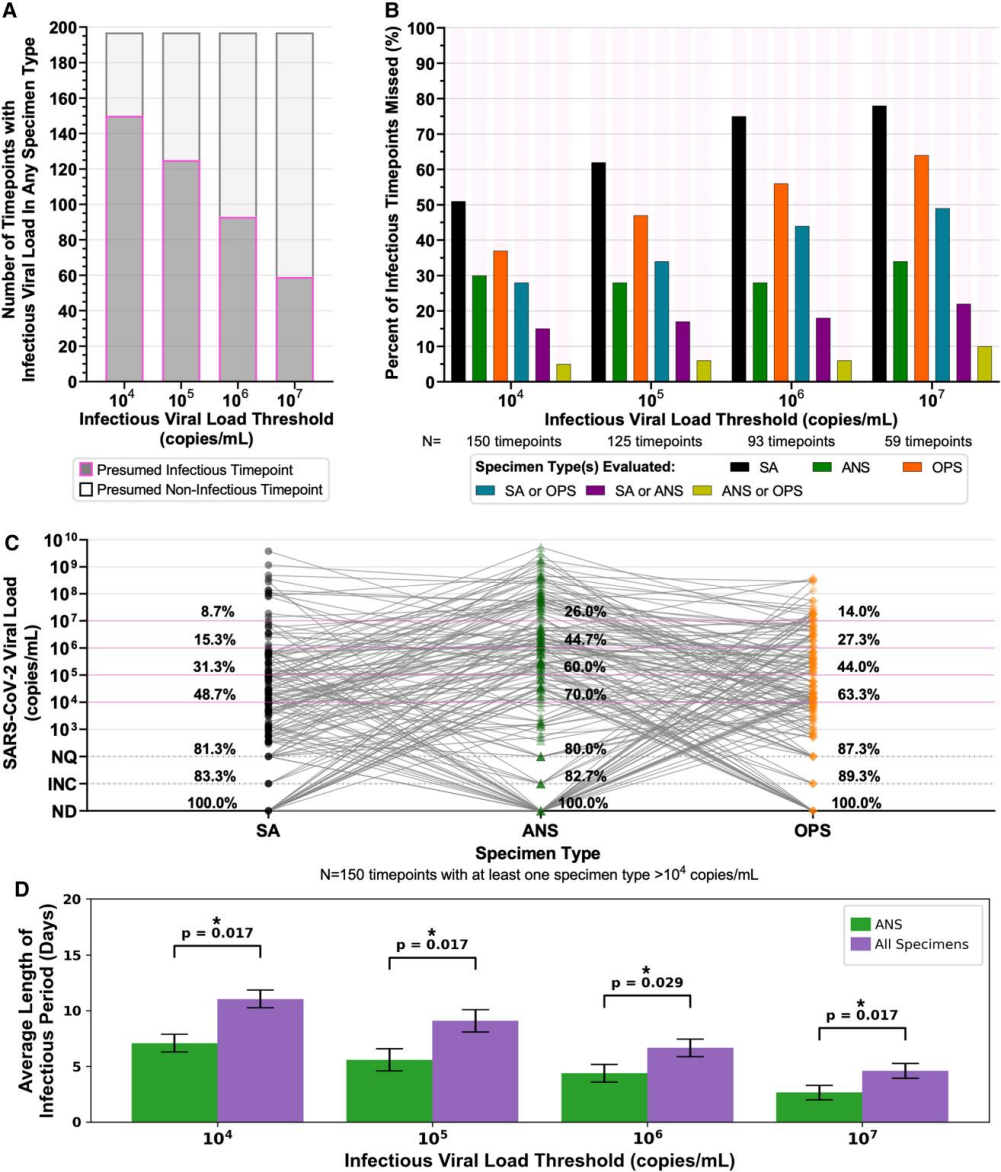
Analyses of presumed infectious viral loads in each specimen type using different infectious thresholds. A) Stacked bar plots of the number of timepoints with at least one specimen type above the indicated infectious viral-load threshold (dark grey with magenta outline), and where all paired specimen types collected at a timepoint had viral loads below the infectious viral-load threshold (light grey with black outline). B) Each bar represents the proportion of all infectious timepoints (i.e. saliva or nasal swab or throat swab had a viral load above the infectious viral-load threshold), where the given specimen type or combination of specimen types did not have an infectious viral load. For example, with an infectious viral-load threshold of 10^4^ copies/mL, 150 timepoints had an infectious viral load in at least one specimen type: in 105 of those 150 timepoints (70%), the nasal-swab (ANS) specimen had an infectious viral load. Therefore, 30% of infectious timepoints would be missed if only the ANS specimen type were evaluated for infectious viral load. Each group of bars provides values for alternate infectious viral-load thresholds, 10^5^, 10^6^, and 10^7^ copies/mL. C) Viral loads of all three specimen types collected by each participant at the same timepoint where at least one specimen type had a viral load above 10^4^ copies/mL (*N* = 150 timepoints). Percentages above each specimen type provide the cumulative proportion of specimens with viral loads at or above each line. Horizontal magenta lines indicate possible infectious viral-load thresholds based on literature. D) Average length of the infectious period when considering only presumably infectious loads in ANS (green) or when considering all specimen types (purple). Error bars are SEM. *P*-values were obtained by performing related-sample *t*-tests for each IVLT. *P*-values were adjusted using two-stage Benjamini–Hochberg correction to account for multiple hypotheses being tested. ANS, anterior-nares swab; SA, saliva; OPS, oropharyngeal swab; ND, not detected by RT-qPCR; INC, inconclusive result by RT-qPCR; NQ, not quantifiable by RT-qPCR.

**Fig. 6.**
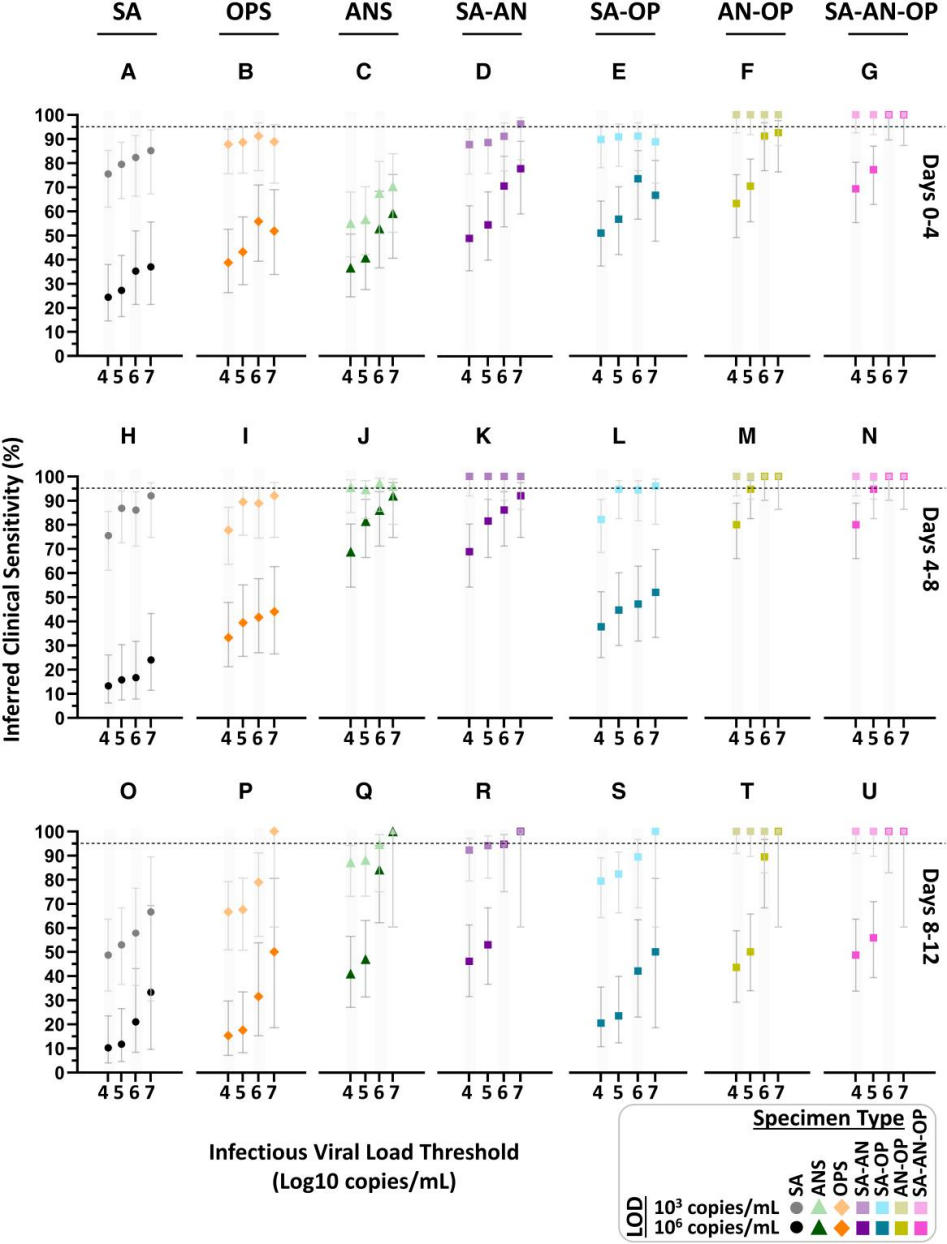
Inferred clinical sensitivity of high- and low-analytical-sensitivity assays to detect presumed infectious individuals by testing single and combination specimen types throughout acute, incident infection. For each 4-day timebin (A–G, H–N, and O–U) relative to the first SARS-CoV-2 positive specimen (of any type), participants were classified as being presumed infectious if viral load in any specimen type collected at a given timepoint was above an infectious viral load threshold. For a high-analytical-sensitivity assay with an LOD of 10^3^ copies/mL and low-analytical-sensitivity assay with an LOD of 10^6^ copies/mL, the inferred clinical sensitivity was calculated as the number of specimens of that specimen type with a measured viral load at or above the LOD divided by the total specimen-collection timepoints included in that timebin. Error bars indicate the 95% CI. The viral load of computationally contrived combination specimen types was taken as the higher viral load of the specimen types included in the combination collected by a participant at a given timepoint. SA, saliva; ANS, anterior-nares swab; OPS, oropharyngeal (throat) swab; SA–AN, saliva-anterior-nares swab combination; SA-OP, saliva–oropharyngeal combination swab; AN–OPS, anterior-nares–oropharyngeal combination swab; SA–AN–OP, saliva-anterior-nares–oropharyngeal combination swab. Inferred clinical sensitivity for LODs from 10^2.4^ to 10^8^ copies/mL shown in Fig. S8; 2-day timebins are shown in Fig. S9.

**Fig. 7.**
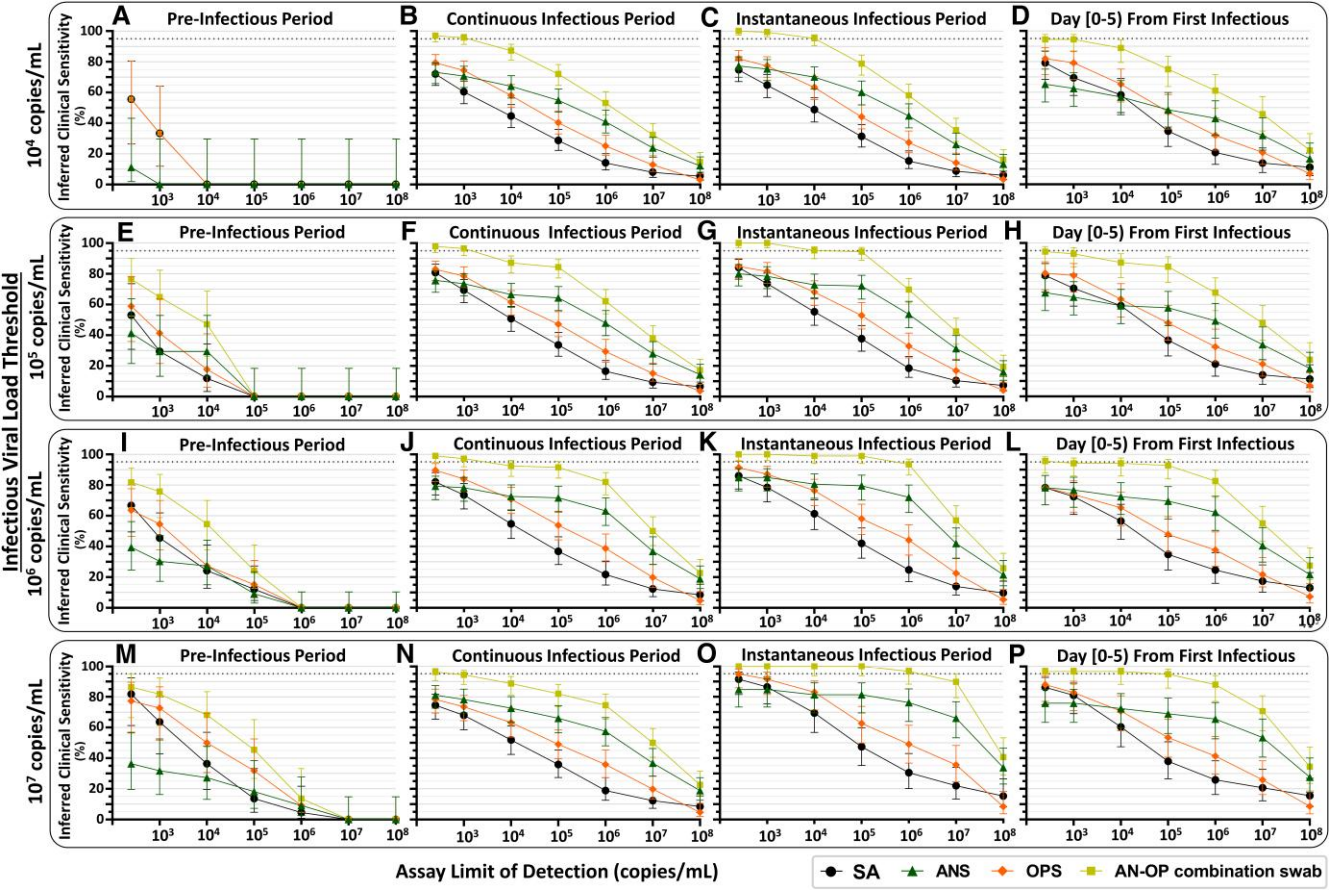
Inferred detection of presumed pre-infectious and infectious individuals at a range of test LODs and with single-specimen tests or AN–OP combination swab specimen type. For each participant, the pre-infectious period was defined as all timepoints with quantifiable SARS-CoV-2 viral load before the first timepoint when at least one specimen type had a viral load above the indicated infectious viral load threshold. We then used three different, common definitions for the infectious period, to assess the robustness of our conclusions. First, we used a “continuous infectious period” whereby a participant is presumed infectious for all timepoints between the first specimen with an infectious viral load and the first timepoint after which no specimens had infectious viral loads. Second, we used an “instantaneous infectious period,” which presumes that a participant is infectious only at timepoints when viral load in at least one specimen type is above the infectious viral load threshold. Third, we presumed that a participant is infectious only for the first 5 days from their first timepoint when at least one specimen type had a viral load above the infectious viral load threshold. These three types of infectious periods were determined for each infectious viral-load threshold: 10^4^, 10^5^, 10^6^, and 10^7^ copies/mL. Each panel provides the inferred clinical performance to detect pre-infectious or infectious individuals, using a given specimen type, for a given assay LOD. Inferred clinical sensitivity was calculated as the number of specimens of each type with a viral load above the assay LOD, divided by the total number of specimens of that type in that period of infection. *N* indicates the total number of specimens of each type included in the inferred clinical sensitivity calculation. Dotted line indicates 95% inferred clinical sensitivity. SA, saliva; ANS, anterior-nares swab; OPS, oropharyngeal swab; AN–OP combination swab, predicted combined anterior-nares–oropharyngeal swab specimen type.

### Statistical analyses

#### Comparison of viral-load timecourses across specimen types

To quantify the difference between viral-load timecourses, we first aligned each timecourse to the time of collection of the first SARS-CoV-2-positive specimen (of any type) for each participant. Differences between viral loads from the same infection timepoint were quantified (Fig. 3A, B). We compared both intra- and interparticipant viral-load timecourses: when the lengths of two participant timecourses differed, the longer timecourse was truncated. We then hypothesized that if the viral-load timecourses followed the same time-dependent distribution, then the observed noise between these viral-load measurements would be attributable to expected sampling noise.

Expected sampling noise was estimated as a zero-centered normal distribution fitted on human *RNase P* control target measurements (Fig. S4B, Supplemental Information). The distribution of observed noise was obtained by performing maximum likelihood estimation on each pair of viral-load timecourses being compared (Fig. 3C). We then tested whether observed differences in viral load across pairs of viral-load timecourses could be explained by expected sampling noise alone. *P-*values were obtained from upper-tailed Kolmogorov–Smirnov tests of the differences between the distributions of the observed noise across viral-load timecourses and expected sampling noise. Two-stage Benjamini–Hochberg correction was used to limit the false-discovery rate to 5%; viral-load timecourse comparisons with adjusted *P*-values <0.05 were considered statistically significantly different (Fig. 3D, see Supplemental Information). Analyses were performed in Python 3.8 using the scipy package (64).

#### Inferred clinical sensitivity by viral-load quantification

Inferred clinical sensitivity of each specimen type and analytical sensitivity were calculated for each timebin as the number of specimens of a given type with viral load above a given LOD divided by all participants considered infected (Fig. 4) or infectious (Figs. 6 and 7) at that timepoint. Confidence intervals were calculated as recommended by CLSI (65). Statistical testing for differences in inferred clinical sensitivity were performed for paired data (comparing performance at two LODs for one specimen type, or at one LOD for two paired specimen types collected by a participant at a timepoint) using McNemar exact tests, and for unpaired data (comparing the performance of one specimen type at one LOD between infection stages) using Fisher exact tests. Analyses were performed in Python v3.8.8.

Participants were considered infected from the time of collection of the first SARS-CoV-2-positive specimen (any type) until negative in all three specimen types by RT-qPCR. Individuals were presumed infectious when viral load in any specimen type was above an IVLT (10^4^, 10^5^, 10^6^, or 10^7^ copies/mL).

Combination specimen types were computationally contrived to have either the maximum (Figs. 5–7) or average (Fig. S7) viral loads from the specimen types included in the combination that were collected by a participant at that timepoint.

## Results

Among the 228 participants, incident SARS-CoV-2 infection was observed in 14 participants (Fig. 1), all of whom were enrolled before or at the start of acute SARS-CoV-2 infection with the Omicron variant of concern. All 14 had received at least one vaccine dose >2 weeks prior to enrollment (Table S1). From this cohort, 260 saliva, 260 oropharyngeal (throat) swab, and 260 anterior-nares (nasal) swab specimens were collected for viral-load quantification and plotted relative to enrollment in the study (Fig. 2). All participants additionally took daily rapid antigen tests; analyzed in a separate manuscript (55).

Viral-load timecourses in the earliest stage of acute SARS-CoV-2 infection differed substantially among specimen types and participants (Fig. 2). In only 2 (Fig. 2A, I) of the 14 participants, viral loads became quantifiable in all three specimen types at the same timepoint; in most (11 out of 14) participants (Fig. 2B, C, D, E, F, G, H, J, K, L, M), saliva or throat swabs were positive first, while nasal swabs remained negative or at low, inconsistently detectable viral loads for up to the first 6–7 days of infection. However, later in the infection, peak viral loads in nasal swabs were significantly higher than in saliva or throat swabs (Fig. S1).

Surprisingly, several participants reported zero symptoms on the day of their peak viral loads (Fig. 2A, C, G, N), all of which were >10^6^ copies/mL. Overall, we found only a weak relationship between viral load and symptoms (Fig. S1D). Importantly, individuals had infectious viral loads in 42% of timepoints at which no symptoms were reported (Fig. S1E).

## SARS-CoV-2 viral loads differ significantly between specimen types during the early period of infection

We next sought to quantify the magnitude of differences in viral load across paired specimens, to answer three questions: (i) Are differences in viral loads between specimen types large enough to impact detectability by assays with varying analytical sensitivity? (ii) Are differences in viral loads attributable to variability in participant sampling behavior? (iii) Are viral-load timecourses in different specimen types within a person correlated with each other?

First, we calculated the absolute (Fig. 3A) and relative (fold) differences (Fig. S3) in viral loads between paired specimens of different types collected by the same participant at the same timepoint. Large differences in absolute viral loads were observed between paired specimen types for both the first 4 days from the incidence of infection (Fig. 3A) and all timepoints (Fig. 3B). We observed absolute differences of >9 orders of magnitude, and all specimen type comparisons had median absolute differences greater than 10^4^ copies/mL, a scale of difference likely to impact the detection of SARS-CoV-2.

If the observed differences in viral loads between specimen types were the result of variability in sample collection during self-sampling, we would expect the fold differences to be similar to the variability of the human *RNase P* control marker. However, *RNase P* Ct measurements were relatively stable for each specimen type collected by participants across their timecourse (Figs. 2 and S4). For some participants, *RNase P* Ct values decreased slightly after the first sample collection, but the average standard deviation in *RNase P* Ct across all participants was <1.5 in all specimen types (saliva: 1.37, nasal: 1.42, throat: 1.46) over enrollment (Fig. S4B), which corresponds to, at most, a 2.8-fold change in target abundance. In contrast, most (84%) comparisons between specimen types had a >2.8-fold difference in viral load (Fig. S2B), demonstrating the extreme differences in load were not due to variability in self-sampling.

Although the differences in viral loads across paired specimens of different types were extreme, we recognized the possibility that the longitudinal timecourse (the rise and fall of viral loads) from different specimen types in a person might still be synchronized. For example, viral loads in one specimen type (e.g. saliva) might be consistently lower than those in another (e.g. nasal), but follow the same pattern throughout acute infection. If this were the case, viral load measured in one specimen type would still be associated with the viral load in another specimen type despite extreme absolute differences. To test whether timecourses from different specimen types were synchronized, we quantified the correlation between viral-load timecourses for each specimen type collected from a single participant, and across different participants. These intra- and interparticipant correlations are represented as a matrix for the 42 viral-load timecourses (14 participants with three specimen types each; Fig. 3C, D). The strength of each correlation (Fig. 3C) was quantified by estimating the standard deviation of pairwise differences in viral load across the two timecourses. The statistical significance of the correlations between viral-load timecourses (Fig. 3D) was then calculated by comparing the distribution of pairwise differences in viral-load timecourses to a distribution of expected sampling noise.

We found that viral-load timecourses in different specimen types collected by the same individual often do not correlate. In nearly all participants (13 of 14), at least two specimen types from the same participant had significantly different timecourses. In 38% of comparisons (16 of 42), we observed significantly different timecourses for each of the three specimen types from the same individual (Fig. 3D). In some instances, the timecourses of specimen types from the same participant were less correlated with each other than with other participants. For example (see white circles in Fig. 3D), the saliva viral-load timecourse for individual A was not significantly different from the saliva timecourses for participants D, F, G, H, J, K, L, or M; however, individual A's saliva timecourse was significantly different from the participant's own throat timecourse.

Within the same individual, throat-swab and nasal-swab viral-load timecourses were most commonly different (64%, 9 of 14 individuals). Additionally, in 29% (4 of 14) of individuals, saliva and nasal-swab viral-load timecourses differed significantly. Finally, despite the proximity of the two oral sampling locations in 21% (3 of 14) of individuals, their own saliva and throat viral-load timecourses were significantly different (Fig. 3D).

## Clinical sensitivity to detect SARS-CoV-2 infection strongly depends on infection stage, specimen type, and assay analytical sensitivity

Because viral load determines whether an assay with a given analytical sensitivity will reliably yield a positive result, we hypothesized that the extreme differences in viral loads among different specimen types would significantly impact the clinical sensitivity of COVID-19 tests performed on different specimen types during different stages of the infection. To examine the inferred clinical sensitivity to detect SARS-CoV-2 infections as a factor of both specimen type and test LOD, viral-load timecourses were aligned to first detectable viral load and divided into 4-day timebins. We assumed that only viral loads above a given assay's LOD would reliably yield a positive result. The inferred clinical sensitivity of detecting infected persons by each specimen type and assay LOD during each timebin was calculated as the proportion of specimens with viral loads greater than the assay LOD, divided by all timepoints collected by infected participants in that same timebin (Fig. 4).

For all specimen types and timebins, testing with a high-analytical-sensitivity assay (LOD of 10^3^ copies/mL) yielded significantly better inferred clinical sensitivity to detect infected persons than testing with a low-analytical-sensitivity assay (LOD of 10^6^ copies/mL; Table S4A–I). During the first 4 days of infection, when individuals are often pre-symptomatic, no single-specimen type achieved >90% inferred clinical sensitivity with any LOD (Fig. 4A–C), suggesting that no single-specimen type will reliably provide early detection of infection with the Omicron variant.

In the first 4 days, nasal swabs generally had the poorest inferred clinical sensitivity of all three specimen types. Even with a high-analytical-sensitivity assay (LOD of 10^3^ copies/mL), nasal swabs were predicted to miss more than half (54%) of timepoints from infected persons. Saliva and throat-swab specimens had significantly better inferred clinical sensitivity than nasal swabs when a high-analytical-sensitivity assay was used, and worse (but not significantly) when a low-analytical-sensitivity assay (LOD of 10^6^ copies/mL) was used (Table S4J–Z).

As infection progresses to days 4–8, individuals are more likely to become symptomatic. Inferred nasal-swab performance improved significantly during days 4–8 (Table S4AH–AN) and became significantly better than saliva and throat swabs at LODs of 10^3^ copies/mL and above (Table S4AO–BB). This improvement can be attributed to the rise to high viral loads in nasal swabs during this period: SARS-CoV-2 was not detected in almost half of nasal swabs in days 0–4, but in days 4–8, more than half of nasal swabs had high viral loads (>10^6^ copies/mL; Fig. S1I, J). In contrast, during both timebins, more than half of all saliva or throat-swab specimens had viral loads below 10^6^ copies/mL, and thus detection using saliva or throat swabs was more dependent on assay LOD (Fig. S1I, J).

## Differences in viral loads among specimen types hinders detection of presumably infectious individuals when tests utilize single specimen types

Prompt identification of individuals who are or will become infectious can prevent further transmission. We next compared the ability of each specimen type and assay analytical sensitivity to detect presumably infectious individuals. An individual was presumed to be infectious if the viral load in any specimen type collected from that participant at a given timepoint was above an infectious viral load threshold. We performed separate analyses for four well-accepted infectious viral-load thresholds (log values of 10^4^–10^7^ copies/mL) to test the robustness of our conclusions.

We found that because of the extreme differences in viral-load timecourses, a presumed noninfectious viral load in one specimen type did not reliably indicate that a participant would have presumed noninfectious viral loads in all specimen types. At the highest infectious viral-load threshold (10^7^ copies/mL), a presumed noninfectious viral load in one specimen type (Fig. 5A) correctly inferred the participant did not have an infectious viral load in any specimen type collected at that timepoint 70% of the time (138 of 197 timepoints). In contrast, at the lowest infectious viral-load threshold (10^4^ copies/mL), a presumed noninfectious viral load in one specimen type correctly inferred a noninfectious participant only about 24% of the time (47 of 197 timepoints).

Across infectious viral-load thresholds, we saw a pattern that suggested combination specimen types might capture more presumably infectious timepoints than single-specimen types (Fig. 5B and C), as 90–95% of timepoints with a presumed infectious viral load in any specimen type had infectious viral loads in either nasal swab or throat swab. This complementarity suggested that a nasal–throat combination swab could be superior for detecting nearly all infectious timepoints.

We interrogated this complementarity between nasal and throat swabs by comparing the viral loads of the three specimen types at each of the 150 timepoints in which at least one specimen had viral loads above a 10^4^ copies/mL infectious viral-load threshold (Fig. 5C). We found that 52% of individuals with presumed noninfectious viral loads in saliva, 38% of individuals with presumed noninfectious viral loads in throat swabs, and 30% of individuals with presumed noninfectious viral loads in nasal swabs actually had presumably infectious viral loads in another specimen type at the same timepoint. In some cases, high-analytical-sensitivity testing could capture individuals with infectious viral loads in specimen types other than the one tested. However, 19% of saliva, 20% of nasal swab, and 13% of throat swab specimens had either undetectable or unquantifiable viral loads while another specimen type in the same individual had presumably infectious viral load (Fig. 5C). In such cases, testing a single-specimen type even with a very-high-analytical-sensitivity assay (e.g. LOD of 250 copies/mL) would not reliably detect a presumably infectious person.

Given that the infectious periods for different specimen types were often asynchronous, considering infectiousness in all three specimen types yielded a significantly longer infectious period than if only nasal viral loads were considered (Fig. 5D) across all infectious viral load thresholds. We also found that the infectious period in nasal swabs and throat swabs together was longer than any other combination of two specimen types, and similar to that of all three specimen types. These results suggest that testing only single-specimen types (such as nasal-swab) may fail to detect individuals with infectious viral loads in untested specimen types.

## Inferring detection of infectious individuals by specimen type and assay analytical sensitivity across infectious viral-load thresholds

Having observed that a person can have low viral loads in one specimen type while having high and infectious loads in another type prompted us to question how well each specimen type and assay LOD would impact the detection of infectious individuals at different stages of the infection. We binned timepoints into 4-day bins and assessed the ability of each specimen type to detect presumably infectious individuals using assays with either high- (LOD 10^3^ copies/mL) or low- (LOD 10^6^ copies/mL) analytical sensitivity in each bin (Fig. 6).

Regardless of specimen type, the inferred clinical sensitivity of both high and low-analytical-sensitivity assays to detect presumed infectious individuals typically increased as the infectious viral-load threshold increased. Improved clinical sensitivity at higher infectious viral-load thresholds was most pronounced for assays with LODs of ≥10^6^ copies/mL. This pattern is intuitive; specimens with viral loads above the infectious viral-load threshold but below the LOD are presumed infectious but missed by the assay, resulting in poor inferred clinical sensitivity. Increasing the infectious viral-load threshold would exclude those specimens from being presumed infectious, thereby resulting in better inferred clinical sensitivity (Fig. 5).

Three major patterns in the specimen types were consistent regardless of the infectious viral-load threshold, so for simplicity the rest of this section describes inferred clinical performances and statistical comparisons using only an infectious viral-load threshold of 10^5^ copies/mL. First, even when tested with a high-analytical-sensitivity assay, no single-specimen type achieved >95% inferred clinical sensitivity to detect presumed infectious individuals (Fig. 6A–C). Second, because the rise in nasal-swab viral load was delayed relative to saliva or throat swab in most participants (Fig. 2), nasal swabs had significantly worse performance than saliva and throat swabs during days 0–4 (Table S4BS–BU). At an assay LOD of 10^3^ copies/mL, the inferred clinical sensitivity of nasal swabs was only 57% (Fig. 6C). This suggests that nasal-swab testing, even with high-analytical sensitivity, would miss ∼43% of presumed infectious individuals the first 4 days of infection. Third, from days 4 to 8 of infection, when nasal-swab viral loads increased rapidly in many participants (Figs. 2 and 4), nasal swabs had significantly higher inferred clinical sensitivity regardless of LOD (Fig. 6C, H–J; Table S4BU, BV) across LODs (Table S4BW–BZ).

## Combination specimen types inferred to significantly improve clinical sensitivity to detect infected and infectious individuals

The extreme differences and lack of correlation in viral loads among specimen types as well as the poor performance of all three specimen types in all timebins and all test LODs led us to hypothesize that combination specimen types might achieve better clinical sensitivity. We generated computationally contrived specimen types representing combinations of specimen types. For each timepoint, the viral load of a combination specimen type was the highest viral load of any single-specimen type included in the combination. We then inferred the clinical sensitivity of these combination specimen types to detect infectious individuals with assays of different analytical sensitivities for each timebin (Fig. 4D–G). The high clinical sensitivity of throat swabs days 0–4, and of nasal swabs at days 4–8 suggested complementarity. Complementarity was further supported by nasal and throat swabs having the most extreme differences in viral load (Fig. 3A, B), that many individuals had significantly different nasal-swab and throat-swab viral-load timecourses (Fig. 3D). Moreover, rarely did individuals have infectious viral loads in saliva alone (Fig. 5, Table S2).

Indeed, the nasal–throat combination swab had higher clinical sensitivity to detect infected individuals than any single-specimen type, at most LODs (Fig. S6). This nasal–throat combination specimen type (Fig. 6F) was also inferred to perform significantly better than all single-specimen types (Fig. 6A–C) at detecting presumed infectious individuals during the first 4 days of infection, and significantly better than saliva (Fig. 6H, O) and throat swab (Fig. 6I, P) during later stages of infection (Fig. 6M, T, Table S4CA–CJ). In addition, the nasal–throat combination swab had significantly better inferred performance than nasal swabs when tested with a low-analytical-sensitivity assay during days 4–8 (Fig. 6J, M). The combination of all three specimen types (Fig. 6G, N, U) would by definition capture all presumed infectious individuals. However, this combination type never had a significantly higher inferred clinical sensitivity than nasal–throat combination swab (Table S4CM–CR).

## Performance of specimen types and analytical sensitivities in the pre-infectious and infectious periods

For public-health purposes, understanding assay performance during the pre-infectious and infectious periods, rather than in timebins relative to the rarely-captured incidence of infection, is more informative and actionable. Therefore, we next evaluated the performance of each single-specimen type and the nasal–throat combination swab for each assay LOD during the presumed pre-infectious and infectious periods (Fig. 7). To ensure our conclusions were robust, we compared the results of our analysis across three definitions of the infectious period: a “continuous” infectious period, an “instantaneous” infectious period, and a “day [0–5]” infectious period (only the first 5 days after an initial presumed infectious specimen; see Materials and methods).

At all infectious viral-load thresholds above 10^5^ copies/mL, the nasal–throat combination swab had the highest inferred clinical sensitivity of any specimen type to detect pre-infectious individuals (Fig. 7E, I, M). In all cases where the assay LOD was at least 2 orders of magnitude lower than the infectious viral-load threshold, there were >10 detectable specimens available for comparison of inferred clinical sensitivity and nasal–throat combination swab was inferred to perform significantly better than nasal swab alone (Table S4CS–DT). With an infectious viral-load threshold of 10^4^ copies/mL, fewer pre-infectious timepoints were available for analysis. In this case, we see that nasal swabs had very low performance, but no specimen type emerged as optimal (Fig. 7A).

Three additional trends held across all infectious viral-load thresholds and all definitions of the infectious period. First, nasal swabs had similar performance to saliva and throat swabs when testing with high-analytical-sensitivity assays (LODs at or below 10^3^ copies/mL), except when infectious period is defined as the 5 days following the first infectious specimen. This definition selects earlier timepoints, prior to the rise in nasal-swab viral loads (Figs. 2 and 5C) so nasal-swab testing had lower inferred clinical sensitivity to detect both infected (Fig. 4B) and infectious (Fig. 6C) individuals. Second, as noted previously (Fig. 4), nasal-swab performance for the detection of infectious individuals was more robust to differences in assay LOD than saliva and throat swabs because nasal-swab loads tended to be either very low or very high (>10^6^ copies/mL), whereas saliva and throat swabs tended to fluctuate between 10^4^ and 10^7^ copies/mL (Fig. S1D, E). Furthermore, in all but one comparison (Fig. 7D), nasal swabs were inferred to have higher performance than saliva or throat swab alone when tested with lower analytical sensitivity assays (LODs at and above 10^5^ copies/mL). Third, a nasal–throat combination swab always had the highest inferred clinical sensitivity at all LODs.

## Discussion

In 14 individuals enrolled before or at the incidence of acute infection, we observed extreme and statistically significant differences in SARS-CoV-2 viral loads among three common respiratory specimen types (saliva, anterior-nares [nasal] swab, and oropharyngeal [throat] swab) collected at the same timepoint from the same individual. In all 14 individuals, we also observed that the viral-load measurements in different specimen types followed significantly different longitudinal timecourses. These intraparticipant differences were as extreme as those observed between participants (Fig. 3C, D). The differences in viral load resulted in significantly different inferred clinical sensitivities to detect both infected and infectious individuals depending on the infection stage, specimen type, and analytical sensitivity (LOD) of the assay. We conclude that unlike infections where a single-specimen type is typically sampled to test for virus (e.g. HIV in blood), SARS-CoV-2 viral load only describes the state of the specimen type tested, not the general state of the individual's infection. A person can have high and presumably infectious viral loads in one specimen type but low or even undetectable loads in another specimen type at the same time point. Thus, defining infectiousness based on assessment of only one specimen type (32, 33, 35, 37, 38, 66–71) likely underestimates the full infectious period, particularly if only nasal swabs (which typically exhibit infectious viral loads days after oral specimen types) are used. Relatedly, policies guiding isolation time that are based on estimates of the infectious period from a single-specimen type may result in premature release of infectious individuals from isolation. Our results also suggest that field evaluations of diagnostics to detect infectious individuals that use a single-specimen type as the comparator assay (67, 72–78) are likely to overestimate the clinical sensitivity of the test being evaluated. Additionally, consideration of infectiousness in multiple specimen types may further elucidate the mechanism behind interpersonal heterogeneity in SARS-CoV-2 transmission to contacts (including super-spreader events) (79).

Because of the extreme differences in viral-load patterns in the early and pre-infectious periods of infection, of the three specimen types considered here, none is optimal for detecting Omicron. However, nasal swab was the poorest specimen type for detection in the first 4 days of infection. In most participants, we observed a delay in nasal-swab viral loads relative to oral specimens similar to what has been observed previously (14, 22, 80) with earlier SARS-CoV-2 variants. In our study, 12 of 14 participants (86%) were either negative in nasal-swab specimens or had nasal-swab viral loads below 250 copies/mL at the incidence of infection (the first day viral RNA was detected in any specimen type). In 3 of these 12 participants (25%), nasal-swab viral loads were either undetectable or inconclusive for >5 days (Fig. S1B, C, H). Because of the delay in nasal-swab viral loads in the first days of infection, the inferred clinical sensitivity of nasal swabs at the beginning of infection was low (<60%), even with high-analytical-sensitivity assays. Although clinical sensitivity of nasal swabs improves later in the infection, which likely coincides with the period after symptom onset in some individuals, the resulting poor clinical sensitivity of nasal swabs raises concerns about the performance of diagnostic tests that use nasal specimens as well as diagnostic assays that have been validated against reference tests that use only nasal specimens.

Furthermore, we found that low-analytical-sensitivity testing was inferred to have poor performance for early detection of infected individuals, regardless of the specimen type used. High-analytical-sensitivity assays (LODs ≤10^3^ copies/mL) were inferred to improve clinical sensitivity in all specimen types and at all stages of infection. We also found that even with high-analytical-sensitivity testing, none of the three specimen types considered here were optimal for detection of presumed infectious individuals (based on viral-load thresholds of 10^4^–10^7^ copies/mL or greater in any specimen type). Of the three single-specimen types, nasal-swab testing was inferred to miss the lowest proportion of presumed infectious individuals overall; yet nasal swabs still missed at least a quarter of all presumably infectious timepoints because of high viral loads in oral specimen types (Figs. 5–7). The failure to detect presumed infectious individuals was inferred to be even worse when using tests of low-analytical sensitivity. To assess this point directly, daily rapid antigen testing results for a broader cohort from this study population are reported separately (55).

Testing with combination specimen types (e.g. sampling from both the throat and nose) was inferred to yield significantly improved clinical sensitivity to detect both infected (Figs. S6 and S7) and presumed infectious individuals (Figs. 6 and 7) than any single-specimen type, regardless of whether the combination specimen type was assumed to have the maximum or the average viral load of constituent specimen types (Fig. S7). Combination swabs have high acceptability (81) and are already common in many regions of the world. In the United Kingdom, the National Health Service website even states that PCR tests that rely only on nasal swabbing will be “less accurate” than those with a combined nose and tonsil swab (53, 54). The United Kingdom also uses a combination nasal–throat swab for rapid antigen testing. However, despite hundreds of emergency use authorizations that the US FDA has issued for diagnostics that detect SARS-CoV-2 (82), including 280 molecular tests and 51 antigen rapid diagnostic tests, none use a combination specimen type.

Our results explain why studies comparing single and oral-nasal combination specimen types have generally shown that combination specimens are either equivalent (26, 83–87) or superior (88–93) to single specimens. Importantly, in nearly all studies evaluating the use of combination swabs, or evaluating combination swab antigen rapid diagnostic tests using a combination swab RT-PCR as reference (33, 51), sample collection began *after* the onset of COVID-like symptoms and/or *after* an initial positive test (usually by nasal swab); thus, they likely did not sample the earliest days of infection, which is the period when we found the greatest benefit of sampling with saliva or a throat swab. One prospective cohort study that did begin testing early (using pre-symptomatic and asymptomatic close contacts) and used combination oropharyngeal–nasal swabs with an RT-qPCR assay as reference to evaluate two antigen rapid diagnostic tests (40) found a similar clinical sensitivity to detect presumed infectious individuals (∼85–90%) with this combination swab specimen type as what we inferred for a combination swab specimen type based on the viral loads in each specimen type individually tested with a moderate- or low-analytical-sensitivity assay. Additionally, longitudinal viral-load timecourses from the incidence of infection in combination nasal–throat specimens have been obtained for participants in a studied that utilized a similar design (41). This combination swab specimen type likely detected infected individuals, despite the heterogeneity that our data suggest would exist between viral loads in each individual specimen type. Infectious virus was also present in this combination swab specimen type early in the course of the infection, which our data suggest would have been missed if only the nose had been sampled.

We note four main study limitations. First, although this is the most comprehensive study of complete viral loads in multiple specimen types to date, data are from a limited number of individuals and demographics. Obtaining early viral-load timecourses from these 14 individuals required enrollment and daily testing of 228 participants for a total of 6,825 RT-qPCR tests. Future studies for new SARS-CoV-2 variants and new respiratory viruses should ideally involve multi-institution partnerships to enroll a diverse cohort from a broad geographic range. Second, we presumed infectiousness based on viral-load thresholds in three specimen types; we did not perform viral culture on these specimens (and acknowledge that specimen types not collected here could have contained infectious viral loads [94]). Third, other specimen types, such as nasopharyngeal swabs, may exhibit different viral-load timecourses and correlate with other specimen types (10). Finally, Omicron remains a relevant variant more than a year after its emergence, but additional variants will continue to develop and may exhibit different patterns in their viral-load timecourses by specimen type. Similar studies will be needed to identify optimal testing methods for new variants (and emerging respiratory viruses).

Viral loads are used in many clinical and basic-science contexts, including diagnostics, epidemiological models, clinical trials, and studies of human immune response. Our results show that early in SARS-CoV-2 infection, viral load cannot be defined for a person, only for a specific specimen type within a person. Thus, when viral-load studies or viral-detection studies are performed with only single-specimen type, the results should be interpreted while considering the heterogeneity of viral loads across specimen types. Additional quantitative longitudinal studies of differences in viral loads in multiple specimen types starting immediately at the incidence of infection are needed for new emerging variants and new respiratory viruses. In the absence of such studies, combination specimen types and tests with high-analytical sensitivity are likely to be the most robust approaches for earliest detection and for the design of studies seeking to assess infection status or presence of infectious virus.

## Supplementary Material

pgad033_Supplementary_Data

## Data Availability

The data underlying the results presented in the study can be accessed at CaltechDATA: https://data.caltech.edu/records/20223.
