# Supplementary material for: Extreme differences in SARS-CoV-2 viral loads among respiratory specimen types during presumed pre-infectious and infectious periods
Source: PNAS Nexus. 2023 Mar 14;2(3):pgad033. doi: 10.1093/pnasnexus/pgad033 (PMC10013338; doi:10.1093/pnasnexus/pgad033)
Supplement: pgad033_Supplementary_Data [file pgad033_supplementary_data.zip › PNASNEXUS-PNASNEXUS-2022-01191-s01.pdf]

Supplemental Information for:

Extreme differences in SARS-CoV-2 viral loads among respiratory specimen types during presumed pre-infectious and infectious periods

Alexander Viloria Winnett[1†], Reid Akana[1†], Natasha Shelby[1†], Hannah Davich[1], Saharai Caldera[1], Taikun Yamada[2,3], John Raymond B. Reyna[2], Anna E. Romano[1], Alyssa M. Carter[1], Mi Kyung Kim[1], Matt Thomson[1], Colten Tognazzini[4], Matthew Feaster[4], Ying-Ying Goh[4], Yap Ching Chew[2,3], Rustem F. Ismagilov[1*]

Affiliations:
[1] California Institute of Technology, Pasadena, CA, USA
[2] Pangea Laboratory LLC, Tustin, CA, USA
[3] Zymo Research Corp., Irvine, CA, USA
[4] Pasadena Public Health Department, Pasadena, CA, USA

*Corresponding author. Email: rustem.admin@caltech.edu

[†]These authors contributed equally to this report

Contents:

Supplemental Materials and Methods

Study Participants
Extraction and RT-qPCR
Quantification of Viral Load from RT-qPCR Result
Viral Sequencing and Lineage/Variant Determination
Shuffled Viral-Load Timecourses and Data Validations
Estimations of Sample Noise with RNase P
Alternate Viral Load Calculation for Computationally Contrived Combination Specimens

Supplemental Figures S1-S9
Supplemental Tables S1-S5
Author Contribution Statements

Supplemental Materials and Methods

Study Participants

All adult participants provided written informed consent, and minors provided assent and their legal guardian provided written permission. Individuals were eligible for enrollment if someone in their home had recently (within 5 days) become positive for SARS-CoV-2, or if they had a recent known exposure to a person suspected to be SARS-CoV-2-positive. All participants had to be 6 years of age or older and fluent in English.

Extraction and RT-qPCR

Participants packaged their specimens each morning for transport by medical courier to Pangea Laboratories in Tustin, CA, USA. Most specimens were received at the facility within 10 hours of collection; some specimens were received at the facility ~24-48 hours after donation due to transport delays. Most specimens were extracted and run in RT-qPCR within a few hours of arrival to the facility. Extraction and RT-qPCR operators and supervisors (at Pangea Laboratory) were blinded to which participant a specimen originated from, as well as the infection status and test results of participants.

Extraction and RT-qPCR were performed using the FDA-authorized Quick SARS-CoV-2 RT-qPCR Kit .[58] which extracts nucleic acids using the Quick-DNA/RNA Viral MagBead Kit (Zymo Research, Catalog #R2141) followed by amplification of three target regions within the SARS-CoV-2 *N* gene.

A specimen was considered inconclusive if the human *RNase P* Ct value was >40 or not detected. If *RNase P* had a Ct < 40, then for a SARS-CoV-2 *N* gene target Ct value <40 the sample was considered positive. If the SARS-CoV-2 target Ct value was 40-45 it was considered inconclusive, and if >45 or not detected it was considered negative.

Quantification of Viral Load from RT-qPCR Result

To quantify viral load in RT-qPCR specimens, a 9-point standard curve was generated at Caltech using dilutions from a commercial heat-inactivated SARS-CoV-2 particles (BEI Cat. N4-52286 Lot 70034991). To achieve higher concentrations and greater dynamic range in the standard curve, volume from a participant saliva specimen previously quantified to have a viral load of 6.44×10^9 copies/mL[53] was used to generate 4 additional points. Diluted particles or volume from the participant specimen was spiked into pooled matrix from freshly collected SA, ANS, or OPS specimens from SARS-CoV-2 negative donors, collected as described above. Specimens were then shipped to Pangea Laboratories (concentrations blinded) for extraction and RT-qPCR testing. Three of three replicates at 250 copies/mL of specimen were detected, independently validating the reported LOD for the assay.

From the dynamic range of the standard curve (250 copies/mL to 4.50×10^8 copies/mL), the following equations were used to convert RT-qPCR SARS-CoV-2 N gene Ct value to viral load in genomic copy equivalents (copies) per mL of each specimen type:

- Viral Load in copies/mL saliva = $2^{(\mathbf{Ct} - 42.374)/-0.8973}$
- Viral Load in copies/mL buffer for nasal swabs = $2^{(\mathbf{Ct} - 43.050)/-0.9282}$
- Viral Load in copies/mL buffer for oropharyngeal swabs = $2^{(\mathbf{Ct} - 43.903)/-0.9653}$

Positive specimens with viral loads that would be quantified below the assay LOD (250 copies/mL) were considered not quantifiable, as amplification and resulting Ct values become noisy at these very low viral loads.

Viral Sequencing and Lineage/Variant Determination

Whenever possible, we sequenced the putative index case's highest viral load nasal-swab specimens. When this was not possible (e.g., if the index case was not enrolled, or the index case's highest viral load nasal-swab specimen was insufficient for sequencing, or limitations in available specimen volume), we chose an alternate high viral load (viral load $<2 \times 10^4$ copies/mL) nasal or oropharyngeal swab specimen from the index case or a secondary case in the household.

All sequencing was performed by Zymo Research at Pangea Lab using a variant ID detection workflow that closely resembles the Illumina COVDISeq™ NGS Test (EUA).[59,60] In brief, RNA extracted from samples underwent cDNA synthesis using random hexamers according to the manufacturer's recommendation (Illumina, Catalog #20043675).

The SARS-CoV-2 virus genome was amplified using primers designed to tile across the full sequence length as originally described by the ARTICnetwork (https://artic.network/ncov-2019). Amplicons containing the SARS-CoV-2 viral genome fragments were then pooled and subjected to tagmentation to further fragment and tag amplicons with adapter sequences. Adapter-tagged amplicons then underwent a second round of PCR amplification using a PCR master mix and unique index adapters. The indexed libraries were then pooled and cleaned up for downstream sequencing.

Finished libraries were sequenced on an Illumina MiniSeq using a PE 100 bp read configuration to a depth of approximately 100,000 reads per library. Illumina sequence reads were converted from bcl to fastq files, adaptor trimmed, then quality filtered using standard parameters. Variant calls as described by Phylogenetic Assignment of Named Global outbreak LINeages software 2.3.2 (github.com/cov-lineages/pangolin) were made using a custom bioinformatics data analysis pipeline developed by Zymo Research.

Shuffled Viral-Load Timecourses and Data Validations

In addition to controls built into the study design (e.g. specimen have barcodes specific to each specimen type, barcodes are confirmed to be the expected specimen type when packaging specimen-collection materials prior to delivery to participants, participants take and package specimen types in a specific order during each timepoint, and the receiving laboratory assessed arriving specimen for the presence of a swab), we assessed mathematically whether the observed viral loads were likely to come from viral-load timecourses of their designated specimen type, or whether they could have been switched between specimen types. We assessed the correlation between the viral load for a given specimen at a timepoint and either the viral load in the same specimen type or the viral load from a different, randomly selected specimen type at the following timepoint (**Fig S3**), for all measurements. The correlation between viral-load measurements from randomly selected specimen types is significantly different ($P<0.001$) from the correlations between viral-load measurements from the same specimen type (**Fig S3C**). Erroneously assigned specimen types would yield similar ($P>0.01$) correlations for both randomized and non-randomized viral-load timecourses. The analysis showed greater standard deviation for shuffled compared with unshuffled viral-load timecourses, suggesting that all specimens were correctly assigned to specimen type by participants.

Estimations of Sample Noise with RNase P

To estimate expected sampling noise that would affect viral-load measurements in each specimen type, we examined RT-qPCR Ct measurements of the human *RNase P* control target in the same specimen type from each of the 14 participants in this cohort (**Fig 2**; **Fig S4B**). The standard deviation of the *RNase P* Ct was calculated for each timecourse and then averaged over all 14 participants: the average standard deviation of *RNase P* Ct for saliva specimens was 1.37, nasal-swab specimens was 1.42, and oropharyngeal swab specimens was 1.46 (**Fig S4B**). We then used the average standard deviation of *RNase P* Ct across all three specimen types (1.42 Ct) as the overall estimate of sampling noise in all viral-load measurements, which is consistent with the standard deviation (1.7 Ct) of SARS-CoV-2 *N2* gene Ct values in two MT nasal-swab specimens collected immediately in sequence in a separate study.[66]

Alternate Viral Load Calculation for Computationally Contrived Combination Specimens

We recognize that specimen-collection and processing factors (e.g., buffer volumes, type and carrying capacity of swabs), may cause dilution effects that would impact the viral load for combination specimen types. To account for this, we also performed an analysis where the viral load of a computationally-contrived combination specimen was calculated as the average (rather than maximum) viral load of paired single specimen types in each combination (**Fig S7**). Using the average introduced at most a 2- or 3-fold correction for the two- or three-specimen combinations, respectively, because viral loads differed by orders of magnitude (**Fig 3**). Clinical sensitivities of combination specimen types remained similar (**Fig S7I-J**) to those calculated in **Fig 4** and the nasal–throat combination swab remained superior with this alternate calculation (**Fig S7F**).

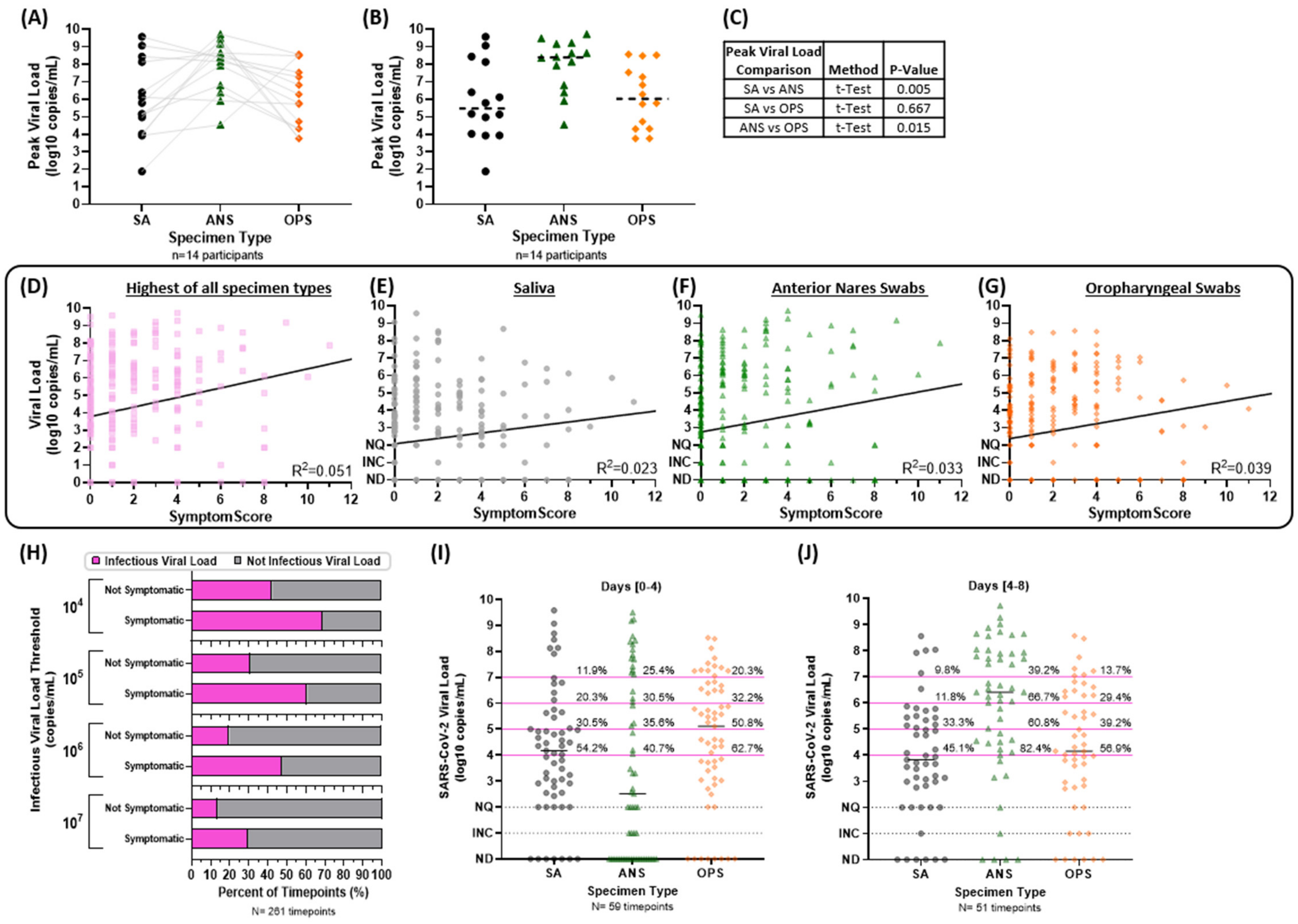

Peak Viral Load Comparison	Method	P-Value
SA vs ANS	t-Test	0.005
SA vs OPS	t-Test	0.667
ANS vs OPS	t-Test	0.015

Figure S1. Peak and distribution of viral loads from the 14 participants enrolled before or at the incidence of acute SARS-CoV-2 infection. (**A**) The peak viral load for each participant is plotted with lines connecting to the viral loads of the other two specimen types at the same timepoint. **(B)** The distribution of peak viral loads for each specimen type is plotted; dashed horizontal bars indicate the medians. (**C**) Table showing statistical test results for comparisons of peak viral load in each specimen type, including the test method, performed in Graphpad Prism 9.2.0. For the cohort of 14 participants enrolled before or at the incidence of infection, the total number of symptoms reported at each timepoint was considered the Symptom Score. The Symptom Score was then plotted against the (**D**) highest viral load in all specimen types, the (**E**) viral load in SA specimens (**F**) ANS specimens and (**G**) OPS specimens. The text on each plot provides the Pearson correlation R squared value, and black lines indicate the line of best fit from linear regression. (**H**) For each symptomatic (Symptom Score >0) or asymptomatic timepoint, viral loads in any specimen type above the given IVLTs were considered infectious (magenta) and those below were considered not infectious (grey). The percentage of infectious and not infectious timepoints, for either symptomatic or not symptomatic timepoints is shown as a horizontal stacked bar graph. (**I**) The distribution of viral loads measured from a positive specimen of each specimen type during the first 4 days and (J) days 4 to 8 from the incidence of infection. N indicates the number of positive specimens of each type (by our high-analytical-sensitivity assay). Percentages above magenta lines to the right of each distribution indicate the fraction of all positive specimen of that type with a viral load at or above that infectious threshold. Black horizontal lines indicate the median viral load for each specimen type. SA, saliva; ANS, anterior-nares swab; OPS, oropharyngeal swab; NQ, below quantifiable; INC, inconclusive; ND, not detected.

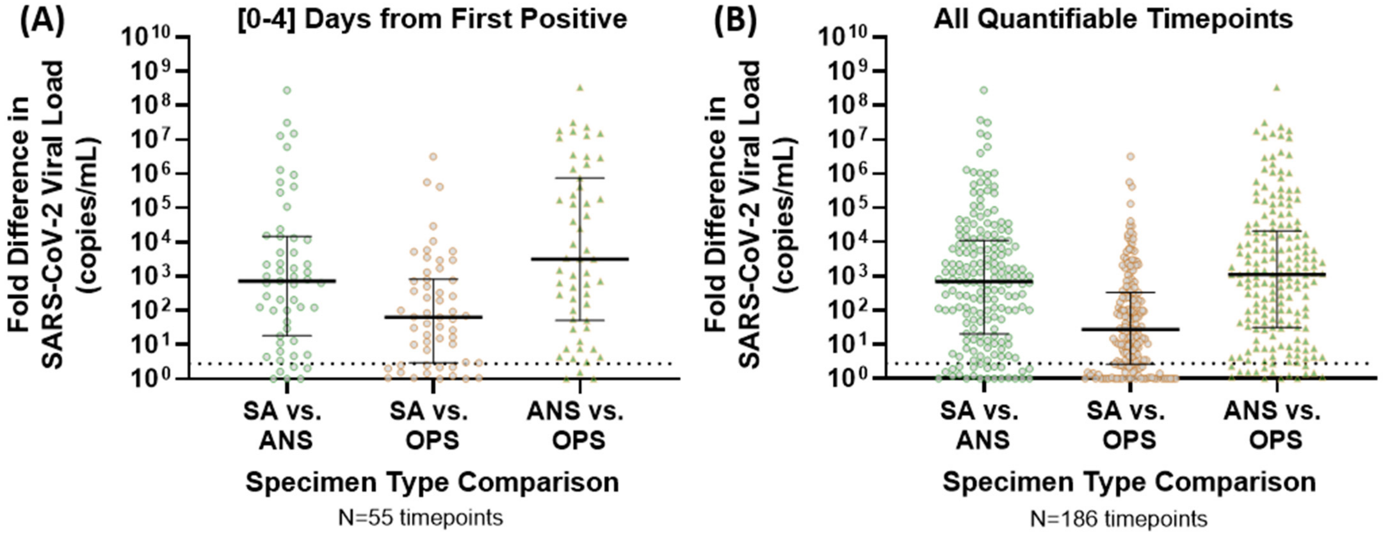

Figure S2. Relative (fold) differences in viral loads from paired specimen types. The fold difference (ratio of higher viral-load specimen of one type over a lower viral-load specimen of another type from the same participant at the same specimen-collection timepoint) are shown for **(A)** the first 4 days of infection (relative to first positive specimen of any type) and **(B)** for specimens collected at all timepoints when at least one specimen from the participant was positive for SARS-CoV-2. Specimens negative for SARS-CoV-2 or with viral loads below quantification had a viral load of 1 copy/mL imputed for calculations. Black bar indicates median. Dashed line indicates 2.8 fold difference, the level of *RNase P* sampling noise (**Fig S4**). SA, saliva; ANS, nasal anterior-nares swab; OPS. oropharyngeal swab, NQ indicates that both specimens being compared had unquantifiable viral loads so an absolute difference could not be calculated.

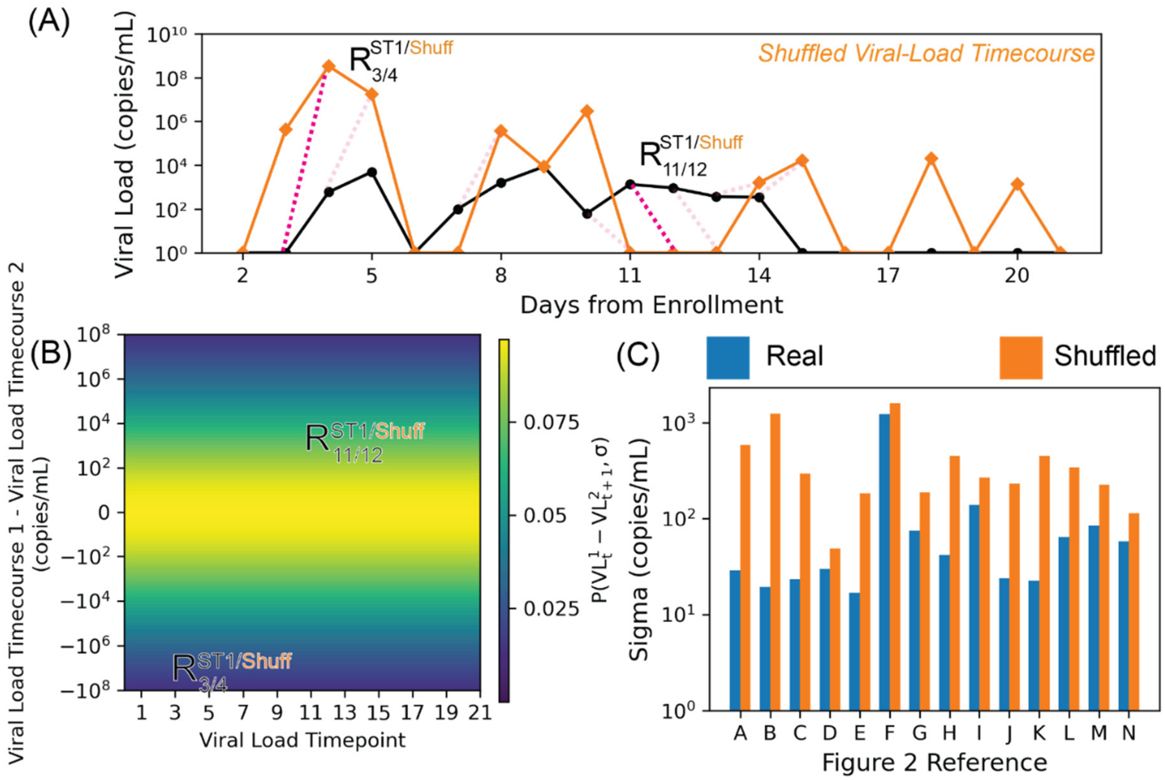

Figure S3. Increased Standard Deviation for Shuffled Viral-Load Timecourses Suggests Correct Sample Assignment by Participant and Specimen Type. (A) Viral-load timecourses for SA specimens collected from participant B (black). A "shuffled" timecourse (orange), obtained by randomizing specimen types at every timepoint, is shown in orange. This "shuffled" timecourse represents data that would be collected if an individual collected the incorrect specimen type when submitting samples. As in Figure S2, differences between timepoints for both "shuffled" and saliva timecourses were calculated. However, the timepoint after the one used for SA is selected for the "shuffled" timecourse. **(B)** Comparisons between pairwise differences between timepoints were visualized on a heatmap. . Background coloring represents the probability of observing pairwise residuals between the shuffled timecourse and the data from the saliva timecourse. Probabilities were generated from a normal distribution centered on 0 with a standard deviation (sigma) generated from the two timecourses. **(C)** Noise obtained from comparison of timecourses against themselves (blue) and shuffled equivalents (orange). Noise was estimated for each of the three specimen types for each individual. Estimates of noise from self-comparisons are statistically significantly from those obtained from comparisons with "shuffled timecourses" (P<0.001).

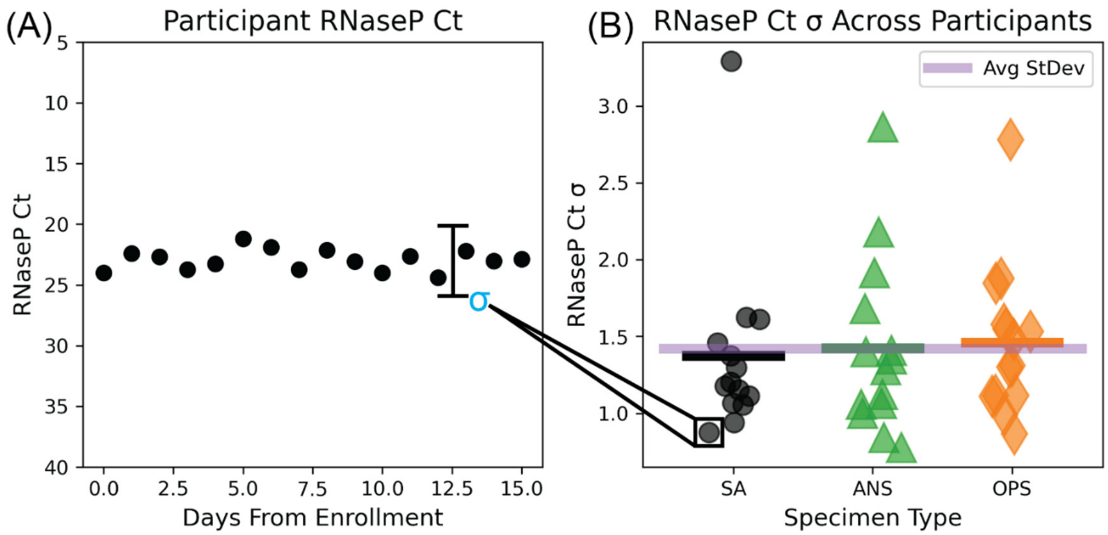

Figure S4. *RNase P* as a Measure of Sampling Variation for 14 Individuals Enrolled At or Before the Incidence of Infection. (A) Example longitudinal *RNase P* Ct measurements from a single individual. σ represents the standard deviation of the *RNase P* timecourse for a single individual in a single specimen type. (B) *RNase P* Ct standard deviations aggregated across specimen types and over all individuals. Horizontal black, green, and orange bars denote average standard deviations for each specimen type (saliva, SA; anterior-nares swab, ANS; oropharyngeal swab, OPS) across participants; the purple horizontal bar represents the average standard deviation over all participants and all specimen types.

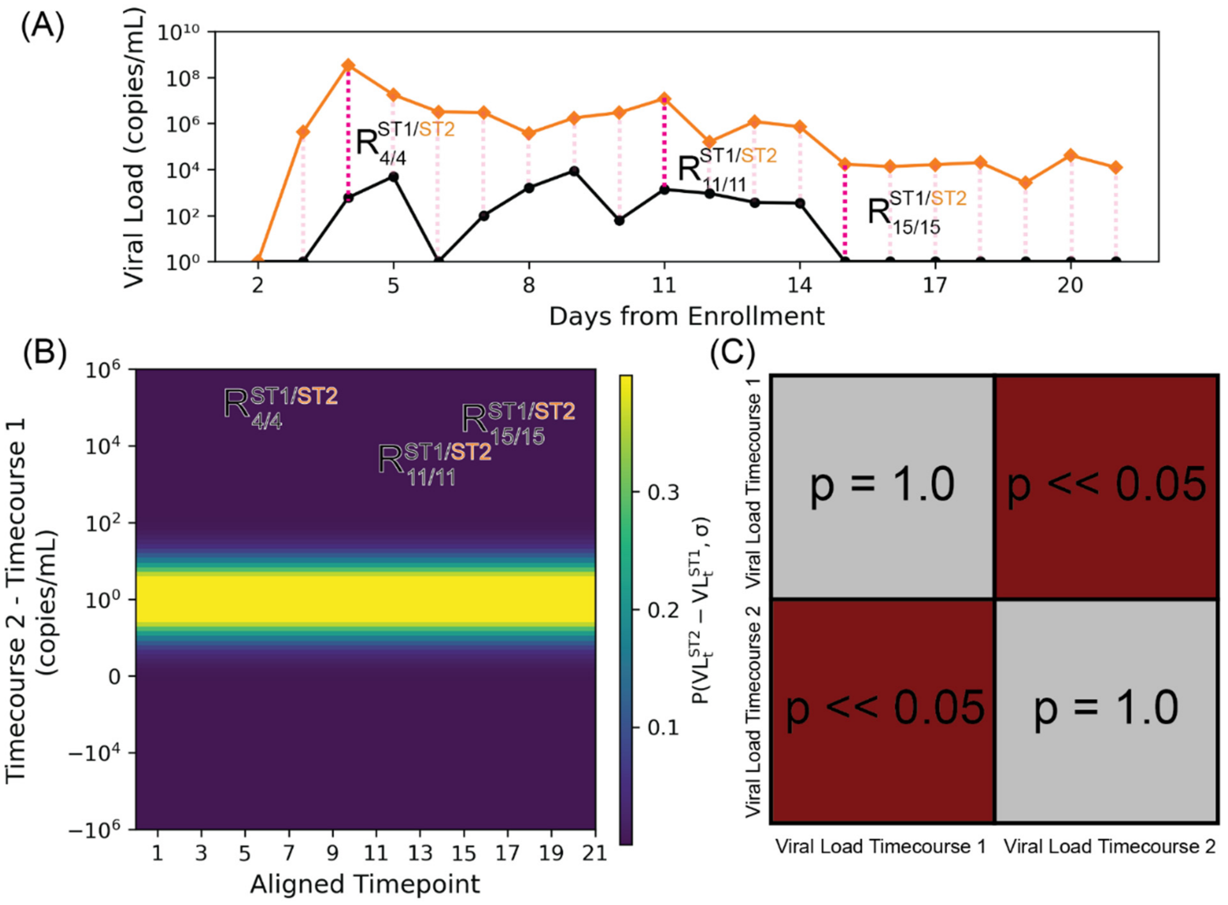

Figure S5. Pairwise Comparison of Viral-Load Timecourses. (A) As an example, the viral-load timecourses for saliva and oropharyngeal swab specimens collected from Z144 are shown. To compare two timecourses, first, the magnitude of the differences between the two timecourses at the same timepoint were calculated. Subscripts refer to time indices and superscripts refer to specimen types. **(B)** These differences were visualized on a graph with the x-axis representing the viral loads of the first timecourse and the y-axis representing the viral loads from the second timecourse. The line y=x, representing perfect agreement between the two timecourses, is plotted in red and background coloring represents probability of observing data given the null hypothesis that the two timecourses are equal. Such probabilities are either estimated from the timecourses themselves (Figure 3A) or from noise contained in *RNase P* data (Figure 3B). **(C)** Statistical significance of differences between viral-load timecourses. Absolute differences between timecourses were compared with the magnitude of bootstrapped noise samples and statistical significance was determined via an upper-tailed hypothesis test. Statistically significant timecourses are depicted in maroon and timecourses that are not significantly different are depicted in gray.

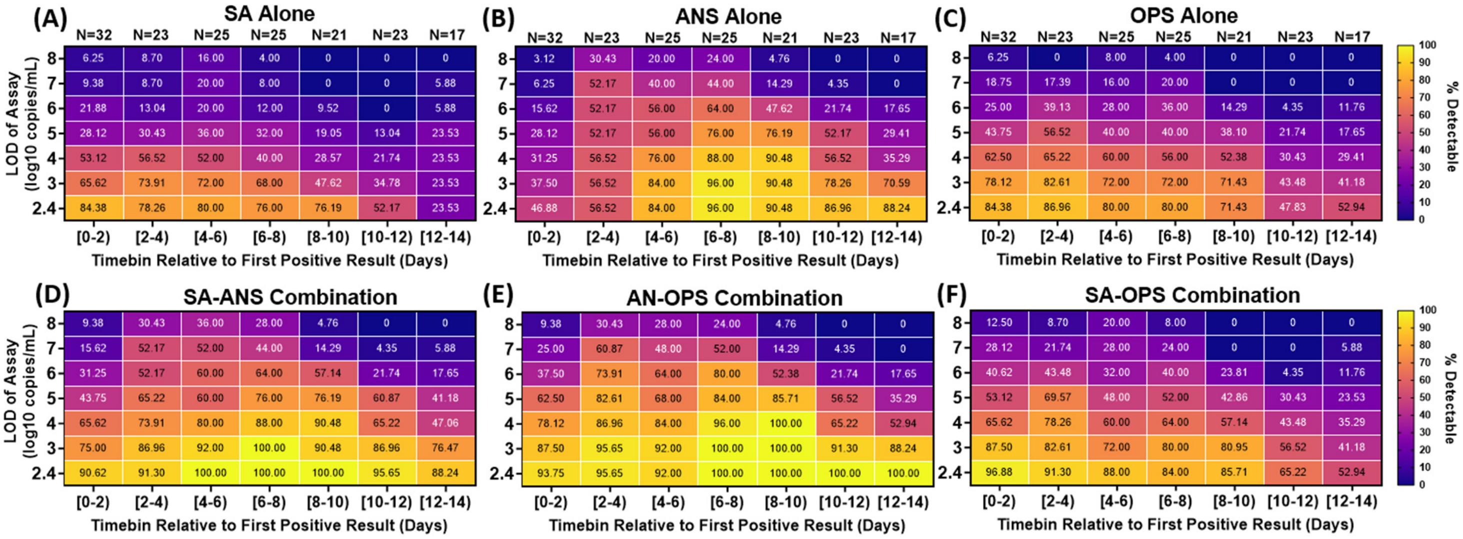

Figure S6. Extreme differences in viral load between specimen types result in low clinical sensitivity to detect infected persons by any single specimen type (A-C) but improved by combination specimen types (D-G). Heatmaps show the inferred clinical sensitivity for (**A**) saliva (SA) specimens alone (**B**) anterior-nares swab (ANS) specimens alone and (**C**) oropharyngeal swab (OPS) specimens alone, throughout the course of the infection (in two-day timebins relative to the first positive specimen of any type) for varying test LODs. Inferred clinical sensitivity was calculated as the number of specimens of the given type with viral loads greater than the given LOD, divided by the total number of specimens collected within that timebin. N indicates the number of specimens for each timebin. Only timepoints where at least one specimen had a quantifiable viral load (250 copies/mL) are included. (**D**) Inferred clinical sensitivity of a computationally-contrived specimen that combines saliva and anterior-nares swab (SA–ANS), **(E)** anterior-nares–oropharyngeal swab (AN–OPS) combination, **(F)** saliva and oropharyngeal swab (SA–OPS) combination, and (**G**) all three specimen types measured. The viral load for these contrived combination specimen types is the higher viral load from the specimen types included in the combination collected by a participant at a given timepoint. SA, saliva; ANS, anterior-nares swab; OPS, oropharyngeal swab. Four-day timebins are shown in **Fig 4**.

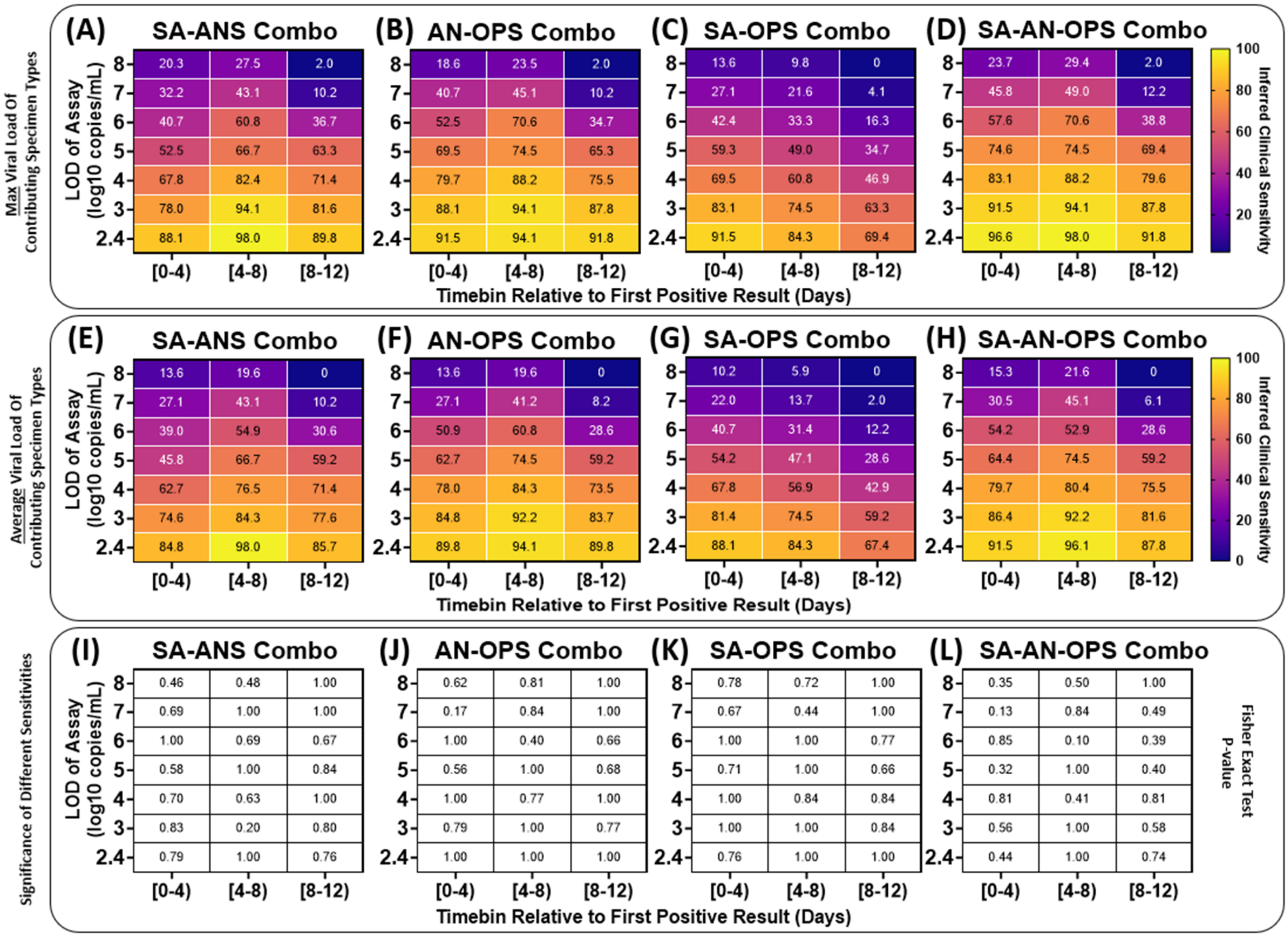

Figure S7. Inferred performance of computationally-contrived combination specimen types by averaging paired single specimen viral loads is similar to taking the maximum viral load of paired single specimen viral loads. Computationally-contrived combination specimen types were generated by taking a function of the viral loads from paired single specimen types collected by a participant at a timepoint. Detection of an infected person was inferred if the viral load in the computationally-contrived specimen type was above the LOD of the assay being used for testing (y-axis). The inferred clinical sensitivity of a given combination specimen type was calculated as the proportion of specimens inferred to be detectable at a given LOD over all positive specimen during each phase of the infection relative to the incidence of infection (x-axis), Each panel provides a heatmap colored by inferred clinical sensitivity when the viral load of computationally-contrived combination specimen types is calculated as the (**A-D**) maximum or (**E-H**) average viral load of paired single specimen types included in the combination, collected by a participant at a given timepoint. The binomial proportions using each function were compared with each other for each cell in each heatmap using the one-sided Fisher Exact Test with the alternative hypothesis that the maximum function would result in greater clinical sensitivity; resulting *P*-values are provided for respective cells in (**I-L**). SA–ANS, saliva–anterior-nares swab combination specimen: AN–OPS, anterior-nares–oropharyngeal swab combination specimen; SA–OPS, saliva–oropharyngeal swab combination specimen; SA–AN–OPS, saliva–anterior-nares–oropharyngeal swab combination specimen.

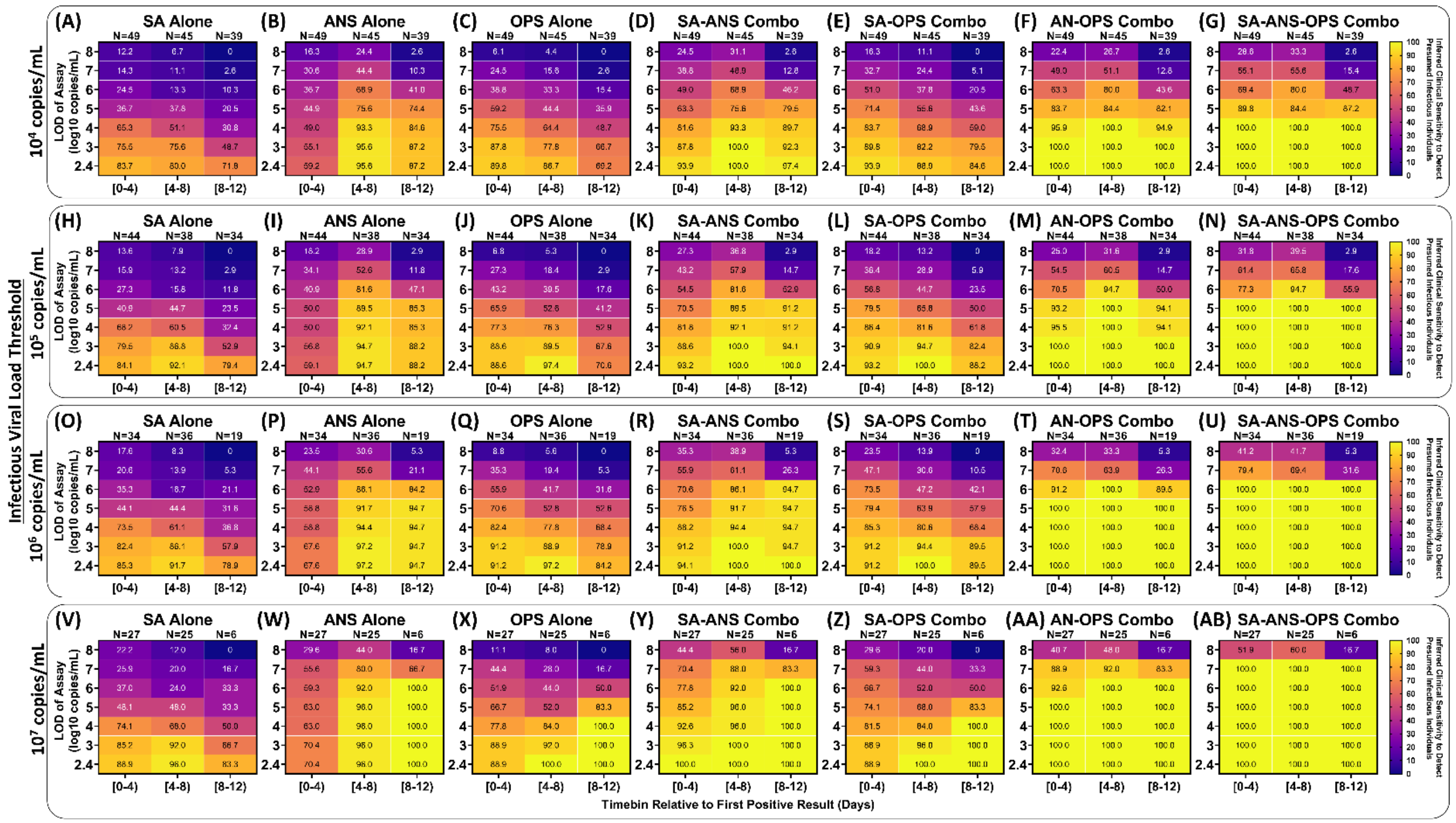

Figure S8. Inferred clinical sensitivity to detect presumed infectious individuals by testing single and combination specimen types using a range of test analytical sensitivities throughout acute, incident infection. For each 4-day timebin relative to the first SARS-CoV-2 positive specimen (of any type), participants were classified as being presumed infectious if viral load in any specimen type collected at a given timepoint was above an infectious viral load threshold (shown on the left side for each group of panels). The inferred clinical sensitivity of each specimen type to detect presumed infectious participants was calculated for each LOD as the number of specimens of that specimen type with a measured viral load at or above the LOD divided by the total specimen-collection timepoints included that timebin. The value inside each cell is the inferred clinical sensitivity to detect a presumed infectious person with that specimen type using an assay with the given LOD during that period of infection. The viral load of computationally- contrived combination specimen types was taken as the higher viral load of the specimen types included in the combination collected by a participant at a given timepoint. SA, saliva; ANS, anterior-nares swab; OPS, oropharyngeal swab. Two-day timebins are shown in **Fig S9**.

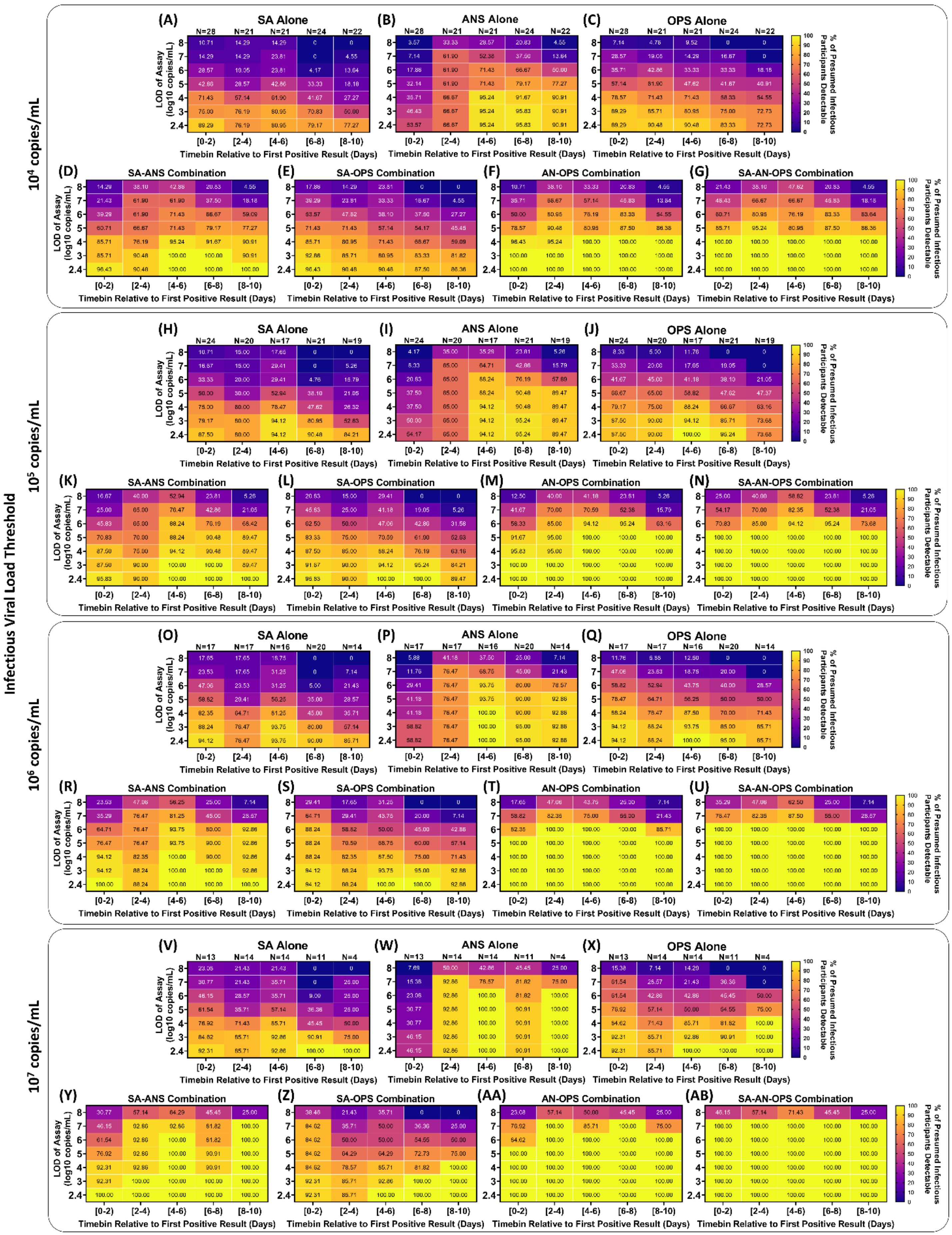
Infectious Viral Load Threshold
10^4 copies/mL
10^5 copies/mL
10^6 copies/mL
10^7 copies/mL
(A) SA Alone
(B) ANS Alone
(C) OPS Alone
(D) SA-ANS Combination
(E) SA-OPS Combination
(F) AN-OPS Combination
(G) SA-AN-OPS Combination
(H) SA Alone
(I) ANS Alone
(J) OPS Alone
(K) SA-ANS Combination
(L) SA-OPS Combination
(M) AN-OPS Combination
(N) SA-AN-OPS Combination
(O) SA Alone
(P) ANS Alone
(Q) OPS Alone
(R) SA-ANS Combination
(S) SA-OPS Combination
(T) AN-OPS Combination
(U) SA-AN-OPS Combination
(V) SA Alone
(W) ANS Alone
(X) OPS Alone
(Y) SA-ANS Combination
(Z) SA-OPS Combination
(AA) AN-OPS Combination
(AB) SA-AN-OPS Combination
LOD of Assay (log10 copies/mL)
Timebin Relative to First Positive Result (Days)
% of Presumed Infectious Participants Detectable

[Figure on prior page] **Figure S9. Inferred detection of presumed infectious individuals by single and combination specimen types and varying test analytical sensitivity throughout acute infection.** For each two-day timebin relative to the first SARS-CoV-2 positive specimen (of any type), participants were classified as being presumed infectious based on whether the viral load in any specimen type collected at a given timepoint was above an infectious viral-load threshold (shown on the left side for each group of panels). The inferred clinical sensitivity of each specimen type to detect presumed infectious participants was calculated for each LOD as the number of specimens of that specimen type with a measured viral load at or above the LOD. The viral load of computationally-contrived combination specimen types was taken as the higher viral load of the specimen types included in the combination collected by a participant at a given timepoint. SA, saliva; ANS, anterior-nares swab; OPS, oropharyngeal swab. Four-day timebins are shown in **Fig S8**.

Table S1. Summary of the demographics, medical information, and vaccine history for the 14-participant cohort. Detailed information by participant can be found in **Table S5**.

**Sex***		
Male	7	50.0%
Female	7	50.0%
**Age**		
6-11	2	14.3%
12-17	1	7.1%
18-29	2	14.3%
30-39	3	21.4%
40-49	4	28.6%
50-59	2	14.3%
**Race**		
White	11	78.6%
Asian or Pacific Islander	1	7.1%
Multiple Races	2	14.3%
**Ethnicity**		
Hispanic	2	14.3%
Non-Hispanic	12	85.7%
**Tobacco Smoker or Vape User History**		
Current	0	0.0%
Former	2	14.3%
Never	12	85.7%
**Active Medications and Supplements**		
Vitamins/Supplements	6	42.9%
Acetaminophen/NSAIDs	3	21.4%
Allergy medications/Antihistamines	2	14.3%
Antibiotics/Antivirals	1	7.1%
**Medical Comorbidities**		
Asthma	1	7.1%
Anxiety or Depression	2	14.3%
Diabetes	1	7.1%
Overweight/Obesity	6	42.9%
GI condition	2	14.3%
**SARS-CoV-2 Vaccination Status**		
Partially Vaccinated	1	7.1%
Completed Vaccination	5	35.7%
Fully vaccinated and boosted	8	57.1%
No SARS-CoV-2 vaccines reported	0	0.0%

*Participants were asked to report both sex at birth and current gender identity; all participants in this cohort responded cis-gender identities to sex at birth

Table S2. The Number of Presumed Infectious Specimens as a Factor of Specimen Type and Infectious Viral-Load Threshold.

Specimen Type(s)	No. Presumed Infectious Specimens (%) by Infectious Viral Load							
	10^4 copies/mL		10^5 copies/mL		10^6 copies/mL		10^7 copies/mL	
**SA** Only	7	(4.4%)	7	(5.3%)	6	(6.5%)	6	(11%)
**SA** & **NS**	6	(3.9%)	8	(6%)	5	(5.4%)	3	(4.3%)
**SA** & **OPS**	15	(8.8%)	8	(6%)	3	(3.2%)	1	(2.9%)
**SA** & **NS** & **OPS**	45	(32%)	24	(20.7%)	9	(9.7%)	3	(4.3%)
**NS** & **OPS**	12	(11%)	14	(15%)	12	(12.9%)	4	(7.1%)
**NS** Only	42	(27%)	43	(31.3%)	41	(44.1%)	29	(51%)
**OPS** Only	23	(13%)	21	(16%)	17	(18.3%)	13	(19%)
**Total**		(100%)	150	(100%)	**93**	**(100%)**	70	(100%)

Table S3. Times from First Positive by Any Specimen Type to First Viral Load Above Infectious Viral-Load Thresholds (IVLT) of 10^4, 10^5, 10^6, 10^7 copies/mL, and to First Timepoint with All Specimen Types Below IVLT.

Figure 2 Reference IVLT = 10^7 copies/mL	Time First Detected from Enrollment (Days)	Time to First Infectious from Enrollment (Days)	Time to Non-Infectious from Enrollment (Days)	Time to Infectious from First Positive (Days)	Time to Non-Infectious from First Positive (Days)
A	5.25	No Samples Above IVLT	No Samples Above IVLT	No Samples Above IVLT	No Samples Above IVLT
B	3.36	4.41	12.4	1.05	9.00
C	0.84	7.31	8.30	6.47	7.46
D	0.92	4.36	18.4	3.45	17.5
E	3.33	No Samples Above IVLT	No Samples Above IVLT	No Samples Above IVLT	No Samples Above IVLT
F	5.41	No Samples Above IVLT	No Samples Above IVLT	No Samples Above IVLT	No Samples Above IVLT
G	15.3	16.4	23.4	1.03	8.07
H	4.27	5.30	15.2	1.02	11.0
I	2.00	5.41	10.5	3.41	8.51
J	0.88	3.36	8.29	2.48	7.41
K	0.77	3.39	9.38	2.62	8.60
L	1.01	2.49	10.7	1.48	9.64
M	0.90	0.90	11.3	0.00	10.4
N	0.86	1.30	5.32	0.44	4.47
Figure 2 Reference IVLT = 10^6 copies/mL	Time First Detected from Enrollment (Days)	Time to First Infectious from Enrollment (Days)	Time to Non-Infectious from Enrollment (Days)	Time to Infectious from First Positive (Days)	Time to Non-Infectious from First Positive (Days)
A	5.25	12.3	18.4	7.07	13.2
B	3.36	4.41	14.3	1.05	11.0
C	0.84	7.31	9.37	6.47	8.53
D	0.92	4.36	29.2	3.45	28.3
E	3.33	10.4	14.4	7.05	11.1
F	5.41	7.42	10.5	2.01	5.11
G	15.3	16.4	24.4	1.03	9.06
H	4.27	5.30	15.2	1.02	11.0
I	2.00	5.41	10.5	3.41	8.51
J	0.88	2.28	10.4	1.41	9.47
K	0.77	1.41	10.4	0.63	9.60
L	1.01	2.49	10.7	1.48	9.64
M	0.90	0.90	11.3	0.00	10.4
N	0.86	1.30	5.32	0.44	4.47
Figure 2 Reference IVLT = 10^5 copies/mL	Time First Detected from Enrollment (Days)	Time to First Infectious from Enrollment (Days)	Time to Non-Infectious from Enrollment (Days)	Time to Infectious from First Positive (Days)	Time to Non-Infectious from First Positive (Days)
A	5.25	7.36	18.4	2.11	13.2

B	3.36	3.36	15.4	0.00	12.0
C	0.84	2.40	10.4	1.56	9.55
D	0.92	3.34	29.2	2.42	28.3
E	3.33	10.4	14.4	7.05	11.1
F	5.41	7.42	10.5	2.01	5.11
G	15.3	16.4	28.3	1.03	13.0
H	4.27	5.30	16.3	1.02	12.0
I	2.00	3.87	10.5	1.87	8.51
J	0.88	1.30	14.3	0.42	13.4
K	0.77	1.41	10.4	0.63	9.60
L	1.01	1.37	12.8	0.37	11.8
M	0.90	0.90	15.3	0.00	14.4
N	0.86	0.86	5.32	0.00	4.47
Figure 2 Reference IVLT = 10^4 copies/mL	Time First Detected from Enrollment (Days)	Time to First Infectious from Enrollment (Days)	Time to Non-Infectious from Enrollment (Days)	Time to Infectious from First Positive (Days)	Time to Non-Infectious from First Positive (Days)
A	5.25	6.29	19.4	1.04	14.1
B	3.36	3.36	21.4	0.00	18.0
C	0.84	2.4	11.3	1.56	10.5
D	0.92	3.34	29.2	2.42	28.3
E	3.33	6.38	14.4	3.04	11.1
F	5.41	7.42	12.5	2.01	7.04
G	15.3	16.4	28.3	1.03	13.0
H	4.27	5.30	16.3	1.02	12.0
I	2.00	2.00	10.5	0.00	8.51
J	0.88	1.30	14.3	0.42	13.4
K	0.77	0.77	11.4	0.00	10.6
L	1.01	1.01	13.7	0.00	12.7
M	0.9	0.90	15.3	0.00	14.4
N	0.86	0.86	8.4	0.00	7.53

[see attached **Table S4.xlsx**]

Table S4. Statistical comparisons of inferred clinical sensitivity drawn from Fig 7. For select comparisons (across specimen types, assay LODs, infection stages/timebins, or IVLTs), the comparison is stated, along with the inferred clinical sensitivity (with 95% Confidence Intervals), statistical method, and significance of the difference. Index is referenced in the main text. Bolded cells in each row indicate the groups being compared. Values under Contingency Table indicate number of specimens. 'Infectious' indicates timepoints from individuals with a viral load in any specimen type above the infectious viral-load threshold listed in parentheses. Test Methods: A- Lower-Tailed McNemar Exact Test, B- Upper-Tailed McNemar Exact Test, C- Two-Tailed McNemar Exact Test, D- Lower-Tailed Fisher Exact Test. SA, saliva; ANS, anterior-nares swab; OPS, oropharyngeal swab; AN–OP, anterior-nares–oropharyngeal combination swab; SA–ANS, saliva–anterior-nares combination specimen; SA–OPS, saliva–oropharyngeal swab combination specimen; SA–ANS–OPS, saliva–anterior-nares–oropharyngeal swab combination specimen.

Table S5. Demographic and Medical Information for the Participants Shown in Fig 3. SARS-CoV-2 variant was determined by ANS swab in all cases except individual (B) who had low ANS viral loads so viral load was sequenced from a throat swab. The variant for participant (I) is inferred from the household index case.

Fig 3 panel	Status on enrollment			Months* since vaccine #			Active Medications	Comorbidities/ medical conditions	Gender**	Age range (in years)	Race	Ethnicity	SARS-CoV-2 Variant
	Saliva PCR	Throat PCR	Nasal PCR	1st dose	2nd dose	3rd dose							
(A)	**neg**	**neg**	**neg**	9 [M]	8 [M]	<2 [M]	n/a	n/a	male	40-49	White	not Hispanic	Omicron BA.1.1
(B)	**neg**	**neg**	**neg**	11 [JJ]	3 [P]	none	PPI, vitamin/ supplement	obesity, GI condition, anxiety or depression	female	30-39	White	not Hispanic	Omicron BA.1.1
(C)	**inc**	**neg**	**neg**	<1 [P]	none	none	acetaminophen	n/a	male	6-11	Multiple Races	not Hispanic	Omicron BA.1.1
(D)	**neg**	**neg**	**neg**	10 [M]	9 [M]	2 [M]	none	obesity	male	30-39	Asian or Pacific Islander	not Hispanic	Omicron BA.1.1
(E)	**neg**	**neg**	**neg**	>11 [P]	<10 [P]	<3 [P]	allergy medication; acetaminophen, antihistamine, dextromethorphan, phenylephrine HCl, doxylamine	obesity	female	30-39	White	Hispanic	Omicron BA.1
(F)	**neg**	**neg**	**neg**	10 [P]	9 [P]	none	vitamin/ supplement	n/a	female	18-29	White	not Hispanic	Omicron BA.1.1
(G)	**neg**	**neg**	**neg**	<2 [P]	<1 [P]	none	vitamin/ supplement	n/a	male	6-11	White	not Hispanic	Omicron BA.1.1
(H)	**neg**	**neg**	**neg**	10 [M]	9 [M]	2 [M]	vitamin/ supplement	n/a	female	40-49	White	not Hispanic	Omicron BA.1.1
(I)	**neg**	**neg**	**neg**	10 [P]	9 [P]	none	antibiotic, vitamin/ supplement	obesity	male	18-29	White	Hispanic	Omicron BA.1.1 (index case)
(J)	*pos*	*pos*	**inc**	9 [M]	8 [M]	<2 [M]	vitamin/ supplement	anxiety or depression	female	40-49	White	not Hispanic	Omicron BA.1.1
(K)	*pos*	*pos*	**inc**	9.5 [M]	8.5 [M]	0.5 [P]	NSAID	n/a	male	40-49	White	not Hispanic	Omicron BA.1.1
(L)	*pos*	*pos*	*pos*	11 [P]	10 [P]	2 [P]	allergy medication, diabetes medication, cholesterol medication	diabetes, high blood pressure, obesity, asthma, sleep apnea, GI condition	female	50-59	Multiple Races	not Hispanic	Omicron BA.1.1
(M)	*pos*	*pos*	**neg**	10 [M]	9 [M]	2 [M]	SSRI	oveweight, anxiety or depression	male	50-59	White	not Hispanic	Omicron BA.1.1
(N)	*pos*	**neg**	*pos*	5 [P]	4[P]	none	none	n/a	female	12-17	White	not Hispanic	Omicron BA.1.1

* Months from vaccine date are given relative to enrollment date
\# Vaccine abbreviations: [P], Pfizer-BioNTech COVID-19 Vaccine (COMIRNATY); [M], Moderna COVID-19 Vaccine (Spikevax); [JJ], Johnson & Johnson
NQ, not quantifiable; viral load was below the test LOD (250 SARS-CoV-2 RNA copies/mL)
** Participants were asked to report both sex at birth and current gender identity; all participants in this cohort responded cis-gender identities to sex at birth

AUTHOR CONTRIBUTIONS (listed alphabetically by last name):

Reid Akana (RA): Collaborated with AVW in creating digital participant symptom surveys; assisted with data quality control/curation with NS, HD, SC; created current laboratory information management system (LIMS) for specimen logging and tracking. Creation of iOS application for sample logging/tracking. Configured an SQL database for data storage. Created an Apache server and websites to view study data. Configured FTPS server to catalog PCR data. Wrote a Python package to access study data. Trained study coordinators on SQL. Troubleshooting and QC of LIMS. Made Fig 3(C-D) and 5D, and SI Figs S3, S4, S5, Table S2, S3, S4, S5, S6. Wrote and edited the manuscript with AVW and NS.

Alyssa M. Carter (AMC): Assisted with the inventory and archiving of >6,000 samples at Caltech; coordinated shipment of samples to Caltech with AER and JRBR; assisted with procurement of antigen tests; assisted with organizing volunteers and making participant kits; assisted AER in developing and implementing QC for participant kits. Provided feedback and edited the manuscript.

Yap Ching Chew (YCC): Primary liaison with Caltech team. Prepared and provided Zymo SafeCollect kits and related materials to Caltech team. Supervised the extraction, PCR, and QC teams at Pangea Laboratory. Sent PCR results daily to Caltech team. Arranged for Pangea team to perform viral-variant sequencing on selected samples; reported results and provided sequencing files.

Saharai Caldera (SC): Study coordinator; recruited, enrolled and maintained study participants with NS and HD; study-data quality control, curation and archiving with RA, NS, HD and MKK; supplies acquisition with AER, NS, HD and MKK.

Hannah Davich (HD): Lead study coordinator; co-wrote participant informational sheets with NS; developed recruitment strategies and did outreach with NS; participant kit creation and co-coordinated kit-making by volunteers with AER; recruited, enrolled and maintained study participants with NS and SC; managed the study-coordinator inventory; study-data quality control, curation and archiving with RA, NS, SC and MKK; supplies acquisition with AER, NS, SC and MKK.

Matthew Feaster (MF): Co-investigator; collaborated with AVW, MMC, NS, YG, RFI on study design and recruitment strategies; provided guidance and expertise on SARS-CoV-2 epidemiology and local trends.

Ying-Ying Goh (Y-YG): Co-investigator; collaborated with AVW, MMC, NS, MF, RFI on study design and recruitment strategies; provided guidance and expertise on SARS-CoV-2 epidemiology and local trends.

Rustem F. Ismagilov (RFI): Principal investigator; collaborated with AVW, MMC, NS, MF, YYG on study design and recruitment strategies; provided leadership, technical guidance, oversight of all analyses, and was responsible for obtaining the primary funding for the study.

Mi Kyung Kim (MKK): Study coordinator (part-time); maintained participants with NS, HD, and SC; study-data quality control, curation and archiving with RA, NS, SC and HD; supplies acquisition with AER, NS, SC and HD; collected contact info for local university/college student health centers for recruitment; assembled Table S5 with NS.

John Raymond B. Reyna (JRBR): Organized sample labeling and short-term storage of all samples at Pangea Laboratories. Arranged shipment of all samples to Caltech team. Assisted with processing of the specimens.

Anna E. Romano (AER): Co-coordinated kit-making by volunteers with HD; implemented QC process for kit-making; participated in kit-making; managed logistics for the inventory and archiving of >6,000 samples at Caltech; supplies acquisition with HD, NS, SC and MKK; assisted with securing funding; compiled Table S3; organized and performed QC on sequencing data. Provided feedback and edited the manuscript.

Natasha Shelby (NS): Study administrator; collaborated with AVW, RFI, YG, MF on initial study design and recruitment strategies; co-wrote IRB protocol and informed consent with AVW; co-wrote enrollment questionnaire and post-study questionnaire with AVW; initiated the collaboration with Zymo and served as primary liaison throughout study; reviewed pilot sampling data and amended instructional sheets/graphics for specimen collections in collaboration with Zymo; co-wrote participant informational sheets with HD; hired, trained, and supervised the study-coordinator team; developed

recruitment strategies and did outreach with HD; recruited, enrolled and maintained study participants with HD and SC; co-developed participant keep/drop criteria with AVW; performed the daily upload, review, and QC of PCR data received from Zymo; made the daily keep/drop decisions based on viral-load trajectories in each household; made all phone calls to alert presumptive positives of their status and provide resources; study-data quality control, curation and archiving with RA, HD, SC and MKK; organized archiving of all participant data and antigen-test photographs; supplies acquisition with AER, HD, SC and MKK; assisted with securing funding; managed the overall study budget; assembled Figs 1-2 with AVW; assembled Table S2; assembled Table S5 with MKK; managed citations and reference library; verified the underlying data with AVW and RA; co-wrote and edited the manuscript with AVW and RA.

Matt Thomson (MT): Assisted with statistical approach and analyses.

Colten Tognazzini (CT): Coordinated the recruitment efforts at PPHD with case investigators and contact tracers; provided guidance and expertise on SARS-CoV-2 epidemiology and local trends.

Alexander Viloria Winnett (AVW): Collaborated with NS, RFI, YG, MF on initial study design and recruitment strategies; co-wrote IRB protocol and informed consent with NS; co-wrote enrollment questionnaire and post-study questionnaire with NS; co-developed participant keep/drop criteria with NS; funding acquisition; designed and coordinated LOD validation experiments; selected and prepared specimen for viral-variant sequencing with NS, YC, and AER; assisted with the inventory and archiving of >6,000 specimen at Caltech with AER and AMC; minor role supporting outreach by HD and NS; minor role supporting kit-making by AER, HD and AMC; verified the underlying data with NS and RA; assembled Figs 1-2 with NS; performed analysis and prepared Figs 4-7, Table S1, Fig S1, S2, S6, S7, S8, S9. Major contributor to the selection of references. Co-wrote and edited the manuscript with NS and RA.

Taikun Yamada (TY): Performed the RT-qPCR COVID-19 testing at Pangea Laboratory.
